# Unraveling the Role of the Stoichiometry of Atomic Layer Deposited Nickel Cobalt Oxides on the Oxygen Evolution Reaction

**DOI:** 10.1002/advs.202405188

**Published:** 2024-07-03

**Authors:** Renée T. M. van Limpt, Mengmeng Lao, Mihalis N. Tsampas, Mariadriana Creatore

**Affiliations:** ^1^ Department of Applied Physics and Science Education Eindhoven University of Technology Eindhoven 5600 MB Netherlands; ^2^ Dutch Institute for Fundamental Energy Research (DIFFER) Eindhoven 5600 HH Netherlands; ^3^ Eindhoven Institute for Renewable Energy Systems (EIRES) Eindhoven 5600 MB Netherlands

**Keywords:** atomic layer deposition, cobalt nickel oxides, electrochemical activation, oxygen evolution reaction, thin film characterization

## Abstract

Nickel cobalt oxides (NCOs) are promising, non‐precious oxygen evolution reaction (OER) electrocatalysts. However, the stoichiometry‐dependent electrochemical behavior makes it crucial to understand the structure‐OER relationship. In this work, NCO thin film model systems are prepared using atomic layer deposition. In‐depth film characterization shows the phase transition from Ni‐rich rock‐salt films to Co‐rich spinel films. Electrochemical analysis in 1 m KOH reveals a synergistic effect between Co and Ni with optimal performance for the 30 at.% Co film after 500 CV cycles. Electrochemical activation correlates with film composition, specifically increasing activation is observed for more Ni‐rich films as its bulk transitions to the active (oxy)hydroxide phase. In parallel to this transition, the electrochemical surface area (ECSA) increases up to a factor 8. Using an original approach, the changes in ECSA are decoupled from intrinsic OER activity, leading to the conclusion that 70 at.% Co spinel phase NCO films are intrinsically the most active. The studies point to a chemical composition dependent OER mechanism: Co‐rich spinel films show instantly high activities, while the more sustainable Ni‐rich rock‐salt films require extended activation to increase the ECSA and OER performance. The results highlight the added value of working with model systems to disclose structure‐performance mechanisms.

## Graphical abstract

Atomic layer deposition offers control over the chemical composition of nickel cobalt oxide thin films, thereby enabling the investigation of their structure‐oxygen evolution reaction (OER) performance relationship. The continuous hydroxide phase formation in nickel‐rich rock‐salt films proceed in parallel with the increase in their electrochemical surface area and OER performance, whereas cobalt‐rich spinel films show a constant OER activity.

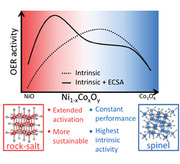

## 1 Introduction

Storage of energy in the form of green hydrogen is a key contributor to a sustainable energy system, with applications for heating, transport and as a feedstock for chemical industry.^[^ ^1^, ^2^ ^]^ Green hydrogen production via intermittent energy sources such as wind and solar energy, requires electrolyzers with suitable dynamic response to electricity fluctuations. Currently, proton exchange membrane water electrolysis (PEMWE) appears to be the most compatible with the intermittent nature of renewable energy due to the employment of a polymeric membrane. The acidic nature of the PEMWE membranes, however, makes the approach reliant on the scarce and expensive platinum group metals (PGMs) which limits broad deployment of electrolysis.^[^ ^1^, ^3^, ^4^ ^]^ The current global annual iridium production, for example, can only support deployment of 3–7.5 GW PEMWE installations per year, whilst a target of 40 GW by 2030 is set for the European Union only.^[^ ^5^ ^]^

The considerations above highlight the need for PGM‐free water electrolyzers. Alkaline water electrolysis is a promising alternative but its liquid electrolyte makes the technique incompatible with the intermittent nature of renewable energy sources.^[^ ^6^, ^7^ ^]^ Anion exchange membrane water electrolysis (AEMWE), however, has the potential to serve this purpose. The technique is based on a polymeric membrane with intrinsic anionic conductivity, such that the alkaline nature of the membrane mitigates the need for PGMs whilst retaining the compatibility with intermittent electricity provision.^[^ ^4^, ^7^, ^8^, ^9^ ^]^ AEMWE devices are still in their infancy and the development of materials and components for the electrolyzer is crucial for its commercialization and widespread adaptation. One important aspect is the design and engineering of the oxygen evolution reaction (OER) electrocatalysts, due to the large overpotential associated with the OER complex four‐electron transfer process.^[^ ^7^, ^9^, ^10^, ^11^ ^]^ In recent years, a wide range of active OER electrocatalysts has been reported.

Transition metal based electrocatalysts are even considered more promising than Ir‐ and Ru‐based catalysts in alkaline media.^[^ ^12^, ^13^ ^]^ Among them transition metal based oxides (TMOs) hold the greatest potential for industrial application, primarily due to the scalability of their synthesis, even though higher catalytic activities have been achieved for other compounds such as (oxy)hydroxides.^[^ ^10^, ^14^, ^15^, ^16^, ^17^ ^]^ First‐row TMOs, such as nickel and cobalt based oxides have attracted particular interest due to their theoretically high efficiency, thermodynamical stability, corrosion resistance and variable chemical reactivity depending on the electronic and crystal structures.^[^ ^12^, ^18^, ^19^, ^20^, ^21^, ^22^, ^23^, ^24^, ^25^, ^26^, ^27^, ^28^ ^]^ Currently, mono‐metallic nickel oxide and cobalt oxide still suffer from limited activity and conductivity. However, the rearrangement of the metal atoms in the binary oxide form more favorable atomic and electronic structures which creates a synergistic effect that improves the OER activity.^[^ ^12^ ^]^ The favorable conductivity of nickel cobalt oxide (NCO) films contributes especially to the interest in AEMWE device level application, as opposed to for example the nickel iron oxides popular in research.^[^ ^8^, ^13^ ^]^

Spinel cobalt oxide (Co_3_O_4_), consisting of Co^2+^ on tetrahedral (Td) sites and Co^3+^ on octahedral (Oh) sites, has been regarded as one of the most active electrocatalysts and is especially renowned for its stability in alkaline conditions.^[^ ^29^, ^30^, ^31^, ^32^, ^33^ ^]^ Improved electrocatalytic performance and electrical conductivity are observed upon the addition of nickel to Co_3_O_4_ such that nickel cobaltite (NiCo_2_O_4_) is formed. This improvement can be related to the formation of an inverse cubic spinel structure, which can be described as (Co^2+^)_Td_[Co^3+^Ni^3+^]_Oh_O_4_, in which Ni cations are situated solely on the octahedral sites whilst Co cations are evenly distributed over the octahedral and tetrahedral sites.^[^ ^25^, ^29^, ^34^, ^35^, ^36^ ^]^ The resulting Ni^3+^‐related unique electronic structure is favorable for the adsorption of ^*^OH intermediates.^[^ ^12^, ^35^ ^]^ NCOs can, however, also structure themselves in a rock‐salt type structure ([Ni^2+^Co^2+^]_Oh_O_2_). Albeit much less popular in research, these binary rock‐salt oxides still show promising electrochemical performances^[^ ^18^, ^23^, ^37^, ^38^, ^39^, ^40^ ^]^ outperforming the more commonly investigated rock‐salt NiO catalyst layer. Opposed to the stable nature of the spinel catalysts, the interest for rock‐salt NiO catalysts originates from its ability to transition into the more active (oxy)hydroxide phase.^[^ ^23^, ^41^, ^42^ ^]^ Additionally, the use of more Ni‐rich catalysts mitigates some social and environmental risks associated with cobalt.^[^ ^43^ ^]^ Investigation of the composition dependent OER activity and activation is therefore crucial for future application of NCO films, given the substantially different benefits of NCO electrocatalysts depending on the stoichiometry.

To the best of the authors’ knowledge, very few reports exist^[^ ^18^, ^23^ ^]^ that systematically investigate the influence of the cobalt atomic concentration, defined as at.% Co = 100% ∙ Co / (Co + Ni), and the corresponding crystal structure, on the electrochemical performance. Trotochaud et al.^[^ ^23^ ^]^ investigated the electrochemical performance of spin‐coated 2–3 nm thick films in 1 m KOH. In their study no synergistic effect was reported between cobalt and nickel, as decreasing at. % Co in the oxide increased the electrochemical performance with an optimal performance for NiO. They suggested that nickel oxide converts into nickel (oxy)hydroxide upon electrochemical activation, and that the presence of cobalt may inhibit this conversion. Deng et al.^[^ ^18^ ^]^ on the other hand, observed an optimal electrochemical performance in 1 m KOH for their ordered mesoporous nickel cobaltite spinel type films, with a synergistic effect between cobalt and nickel and an optimal performance for Ni‐rich spinel type NCOs. These contradicting observations motivate us to further investigate the influence of the cobalt atomic concentration on the OER performance and the mechanism of activation using well‐defined model systems.

Electrocatalysis is a dynamic surface process, where simultaneous changes occurring in chemical composition, structure, and physical properties are considered the main hurdle in understanding structure‐activity relationships.^[^ ^11^, ^44^, ^45^, ^46^, ^47^, ^48^, ^49^, ^50^, ^51^ ^]^ We therefore adopt an atomic layer deposition (ALD) method, which offers the opportunity to prepare well‐defined, conformal, high‐quality film modal systems. ALD consists of a series of self‐limiting half‐cycles based on chemisorption reactions between the selected precursor/co‐reactant and functional groups present at the substrate surface. So far, ALD has already been successfully adopted for deposition of various electrocatalysts.^[^ ^28^, ^52^, ^53^, ^54^, ^55^ ^]^

In our recent work,^[^ ^40^ ^]^ we showed that ALD can be used to prepare smooth, polycrystalline NCO films with tunable chemical composition in terms of Co at.%. Such control leads to the transition of the film crystal structure from a rock‐salt nickel‐rich phase to a spinel type cobalt‐rich phase. Whilst the previous work focused on ALD processing, the present work focuses on the electrochemical behavior of NCO films. Specifically, we investigate the influence of the film chemical composition and crystallographic orientation on the prolonged electrochemical activation of NCO films, such that the intrinsic electrochemical activity can be decoupled from changes in electrochemical surface area (ECSA). The focus of this work is therefore to make an equitable comparison between compositions instead of achieving optimal overpotentials, bearing in mind future application of NCO catalysts in AEMWE. Our investigations highlight the importance of careful selection of the catalyst chemical composition to tune its electrochemical properties, depending on the desired behavior.

To this purpose, first an in‐depth film characterization is provided, which addresses the chemical composition and polycrystalline phase transition of the thin film model systems. Next, the electrochemical performance is evaluated during extended cyclic voltammetry (CV), which reveals a direct correlation between the composition and electrochemical activation, such that rock‐salt films continuously become more OER active whilst a constant performance is observed for spinel films. Chemical analysis of the films shows that the activation is accompanied by the formation of a hydroxide phase, along with an increase in ECSA. Correction of the overpotential through scaling of the current density by the changes in ECSA, allows for decoupling the effect of the ECSA development from the intrinsic activity of the layer. This approach shows how the electrochemical activation mechanism of the NCO films depends on their chemical composition and reveals that nickel cobalt spinel films are intrinsically the most OER active electrocatalysts.

## 2 Results and Discussion

The role of the at.% Co on the electrochemical activation and performance of NCOs has been studied using ALD deposited thin film model systems. The chemical and structural properties of the films are evaluated first using X‐ray Photoelectron Spectroscopy (XPS) and Grazing Incidence X‐Ray Diffraction (GIXRD).

### 2.1 Pristine Film Chemical and Crystallographic Characterization

A series of 20–35 nm NCO thin films depending on the composition is prepared through an ALD process which we published earlier^[^ ^40^ ^]^ and which is highlighted in the experimental section. The films are conformal to the morphology of the FTO glass, with a root mean square (RMS) roughness of 11 ± 2 nm for a 0.5 × 0.5 µm^2^ area as indicated by Atomic Force Microscopy (AFM) (see also Table S1, Supporting Information). Scanning electron microscopy (SEM) (Figure S1, Supporting Information) furthermore confirms the deposition of a smooth film on the FTO substrate.

**Figure 1:**
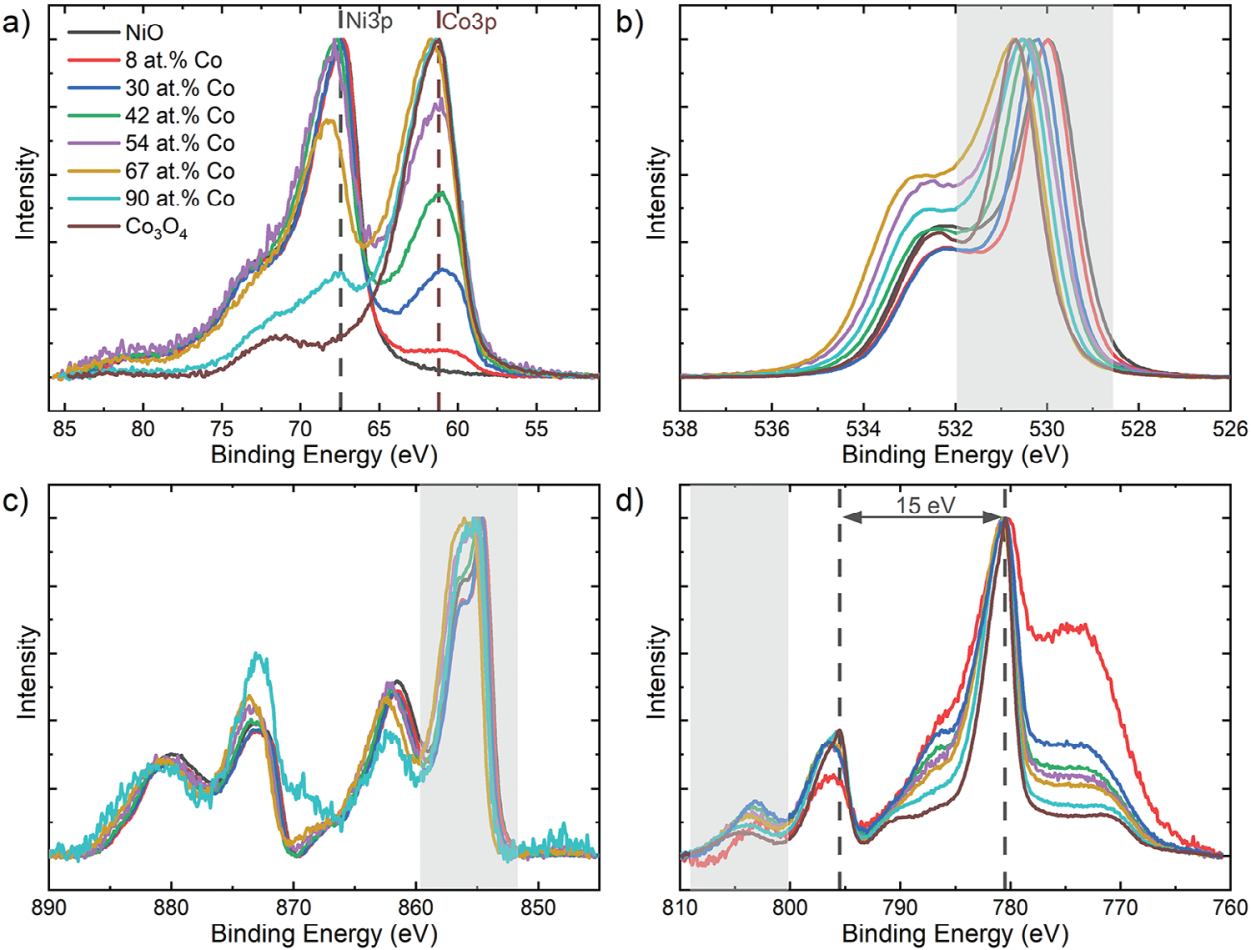
X‐ray spectroscopy measurements at the a) Ni3p and Co3p, b) O1s, c) Ni2p and d) Co2p spectra of the deposited films. The legend of (a) is valid for all figures.

The deposited films contain cobalt ranging from 8 to 91 at.% Co, as inferred by the fitting procedure of the Co3p and Ni3p XPS spectra (**Figure** **1**a) outlined further in Figures S2 and S3 (Supporting Information). Note that the 3p spectra are used for at.% Co determination because loss features of cobalt oxide are present in the Ni2p spectra and vice versa. The Co at.% as determined through the XPS 3p spectra is verified using particle‐induced x‐ray emission (PIXE), where 6.7 ± 0.1 at.% Co is found for a film characterized by XPS to have a cobalt atomic concentration of 6.5 ± 0.3 at.% Co. Figure 1 reports the XPS spectra for several film chemical compositions. All films show a dominant feature at 530 eV with a ≈532 eV shoulder in the O1s spectra (Figure 1b). The main feature confirms the formation of an oxide state, whilst the shoulder is ascribed to chemisorbed water or to adsorbed hydrogen passivated surface oxygen.^[^ ^56^ ^]^ The shoulder is not assigned the formation of a hydroxide as previous quantitative analysis of the hydrogen content in the film using elastic recoil detection shows hydrogen‐to‐oxygen ratios of ≈0.1.^[^ ^40^ ^]^

**Figure 2:**
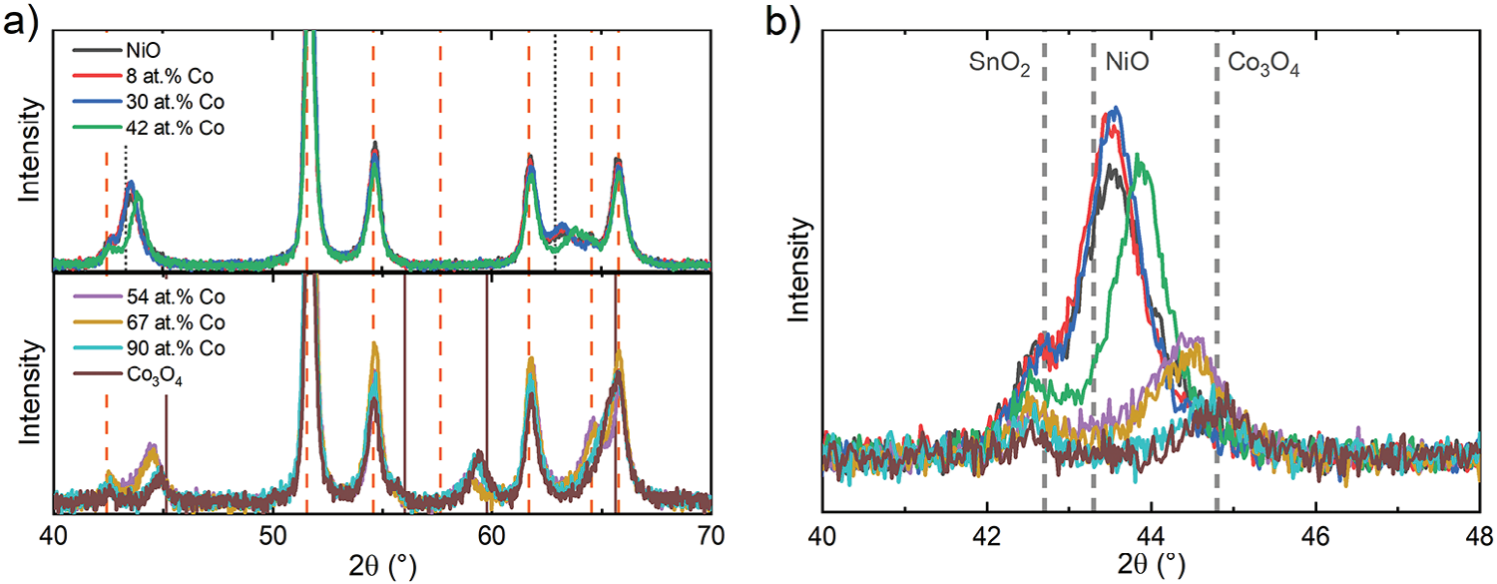
a) X‐ray diffraction pattern of NCO films deposited on FTO glass. The vertical lines refer to the ICSD reference measurements of NiO (grey, dotted), Co_3_O_4_ (brown, solid) and SnO_2_ (orange, dashed) (ICSD cards 9866, 36 256 and 9163, respectively). b) Shift of the angle at 44°. The legend of (a) is also valid for (b).

Information on the metal oxidation states can be extracted from the shaded areas of the Ni2p and Co2p spectra in **Figure** **2** and from the spin orbit splitting of the Co2p spectra (see also Pages S3 and S4, Supporting Information). The Ni2p spectra (Figure 2a) of films up to 30 at.% Co show a dominant feature at 854 eV with a 856 eV shoulder, characteristic of the Ni^2+^ based‐oxide (NiO).^[^ ^57^, ^58^ ^]^ An increase of the feature at 856 eV as compared to 854 eV is observed beyond 30 at.% Co. This increment can be ascribed to an increase in the Ni^3+^‐to‐Ni^2+^ ratio. Note that it is difficult to infer the relative concentration of Ni^3+^ states from the Ni2p spectrum due to mixed contribution of Ni^2+^ and Ni^3+^ states at 856 eV shoulder of the 2p^3/2^ feature, making only a qualitative comparison between films possible. The Co2p spectra (Figure 2b) show a 16 eV spin‐orbit split in combination with significant loss feature around 800–810 eV for films up to 30 at.% Co, which indicates the presence of Co^2+^.^[^ ^59^, ^60^, ^61^ ^]^ For higher concentrations, a decrease in the spin‐orbit split to 15 eV and a decrease in loss feature is observed, indicative of the formation of more Co^3+^ states. These observations align with the expected mixed +2/+3 valence state for Co_3_O_4_.

Next, the crystallographic orientation is investigated using GIXRD (Figure 2). The diffractograms (Figure 2a; Figure S4, Supporting Information) show features at 43° and 63° representative of the polycrystalline rock‐salt orientation below 50 at.% Co. Note that partially overlapping features from the underlying FTO glass substrate hinder observation of some features, such as the 37°‐related rock‐salt feature. Above 50 at.% Co, features are observed at 45°, 59°, and 65°, which are characteristic of the polycrystalline spinel orientation without traces of rock‐salt phase. These observations are in line with our previous results for films deposited on c‐Si.^[^ ^40^ ^]^

The transition from rock‐salt to the spinel structure can be investigated in more detail using the (200) rock‐salt and (400) spinel features between 43°and 45°, respectively (Figure 2b). Within the rock‐salt type films, a significant shift is observed for the (200) feature of the 43 at.% Co film. This shift is attributed to the formation of more +3 oxidation states as observed in XPS. Note that the presence of Co^2+^ in the film cannot explain a shift to higher angles due to its similar ionic radius to Ni^2+^ (0.75 and 0.69 Å respectively^[^ ^62^ ^]^). The (400) features of the spinel films with relatively low Co at.% (57 at.% Co and 72 at.% Co) show a significant shift toward lower angles as compared to cobalt‐rich spinel films. The increase in lattice parameter for the nickel‐rich spinel configuration is attributed to the presence of Ni^3+^ oxidation states, as verified by XPS, which result in the formation of the inverse spinel phase of NiCo_2_O_4_ as compared to the regular spinel phase of Co_3_O_4_.^[^ ^34^, ^35^, ^63^, ^64^, ^65^ ^]^ This assignment can be further confirmed by an increase in electrical conductivity (Figure S5, Supporting Information) to 9.0 × 10^1^ S cm^−1^ observed for the ≈70 at.% Co film, which suggests the formation of an inverse spinel structure as the presence of Ni^3+^ is expected to induce a semi‐metallic state.^[^ ^35^, ^64^, ^66^, ^67^, ^68^ ^]^ In summary, we have deposited smooth NCO films spanning a full range of Co at.%, such that a transition from a rock‐salt film to mixed spinel phase films is observed, which is accompanied by an increase in the transition metal 3+‐to‐2+ oxidation state ratio.

### 2.2 Electrochemical Characterization of NCO Films

The electrochemical performance of the films has been evaluated using CV in high purity 1 m KOH solution prepared from pellets, to provide control over electrolyte‐based Fe impurities especially. First, the influence of the Co at.% on the electrochemical behavior is investigated using the redox couples and the onset potentials. Afterwards, the activation process is evaluated using the overpotential, defined as the difference between the applied potential and the thermodynamic potential of 1.23 V vs RHE, as a function over CV cycles. Note that relatively high overpotentials^[^ ^8^, ^69^ ^]^ are observed due to the use of FTO substrates, which are selected to provide a well‐defined model system.

**Figure 3:**
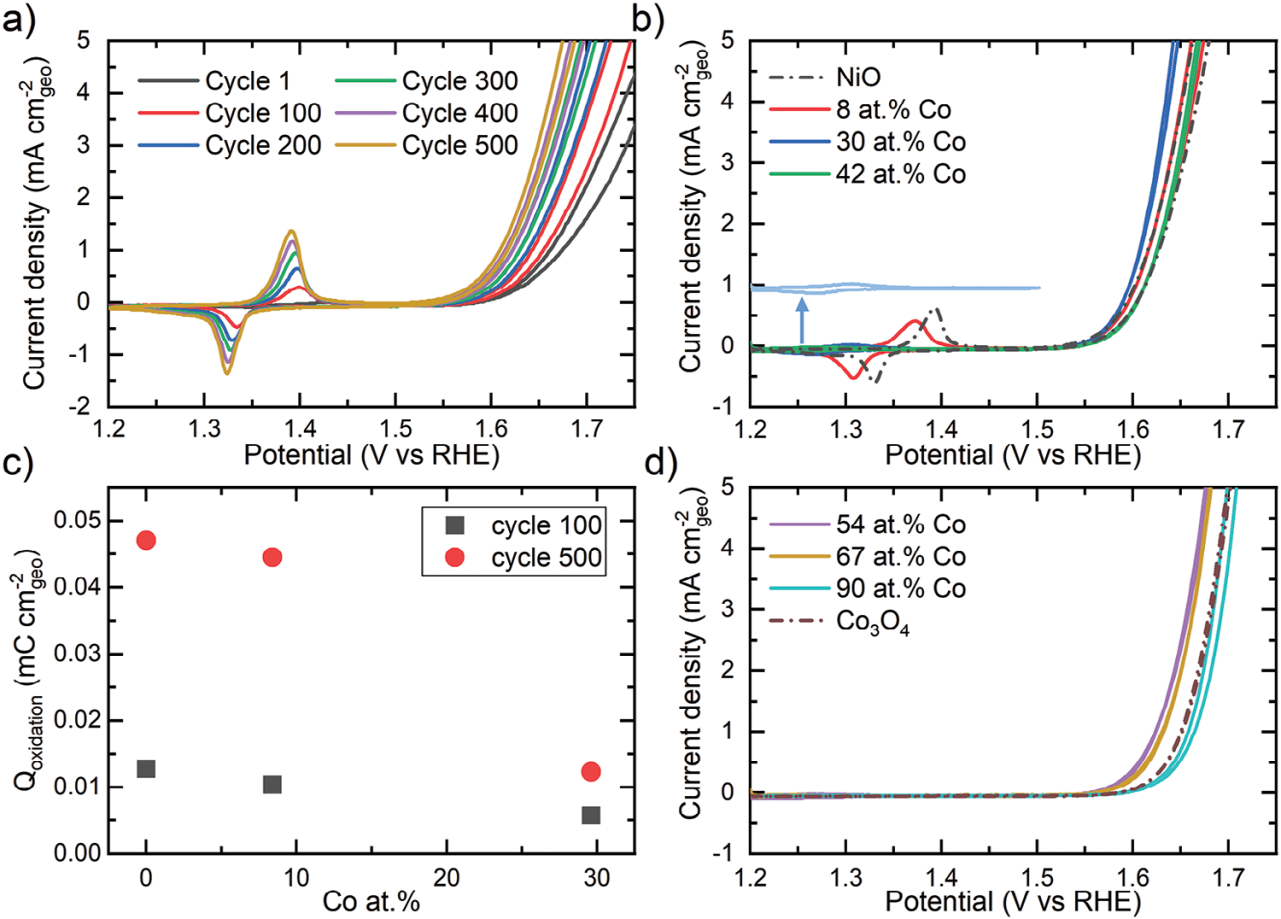
Redox features and onset potential for a) NiO investigated at 50 mV s^−1^; b) rock‐salt films, activated for 500 cycles at 20 mV s^−1^, with different cobalt relative content. The redox feature of 30 at.% Co film is highlighted using an offset. c) The variation in integrated charge, obtained from the 50 mV s^−1^ CV curves after 100 and 500 CV cycles. d) Onset potential of activated (500 cycles) spinel films as a function of Co at.% at 20 mV s^−1^.

The electrochemical behavior of the films is monitored over 500 CV cycles. The NiO film shows the characteristic redox features at 1.39 V vs RHE (oxidation) and 1.33 V vs RHE (reduction) ascribed to the in situ oxidation of Ni(OH)_2_ to the more electrochemically active NiOOH species (**Figure** **3**a).^[^ ^23^, ^28^, ^41^, ^70^, ^71^, ^72^ ^]^ When comparing the electrochemical behavior of NCO rock‐salt films to the of NiO films (Figure 3b) a shift of the redox couple to lower potentials is observed. The shift of redox features as opposed to the formation of two distinct redox waves for Ni(OH)_2_ and Co(OH)_2_ indicates a strong electronic coupling between Ni and Co sites that facilitates the Ni oxidation (and reduction) and can therefore be seen as further evidence of the homogeneous mixing of cobalt and nickel in the oxide phase.^[^ ^23^, ^70^, ^71^, ^73^, ^74^, ^75^ ^]^ Furthermore, these results indicate that there is a synergy between cobalt and nickel that facilitates OER activity. The electrochemical measurements show no significant redox features for the spinel films (Figure 3d) which is in accordance with literature and points toward a surface limited electrochemical activity.^[^ ^18^, ^23^, ^33^, ^76^ ^]^ Simultaneously, the onset potential is observed to shift to higher potentials for spinel films with increasing Co at.%.

More electrochemically active material is formed upon cycling rock‐salt films, as indicated by the increasing redox features and a decrease in the onset potential of the current density (Figure 3a; Figure S6, Supporting Information).^[^ ^23^, ^52^, ^71^, ^73^, ^77^ ^]^ The integrated charge of the rock‐salt redox features is monitored at 100 and 500 CV cycles and shown as a function of Co at.% in Figure 3c. The integrated charge of the NCO rock‐salt films is lower than expected based on the available nickel sites as determined from the cobalt concentration. This can be illustrated by a simple calculation: after 500 cycles a charge transfer of 0.047 ± 0.004 mC cm^−2^_geo_ is observed for NiO. For the 30 at.% Co film, a charge transfer of 0.033 ± 0.003 mC cm^−2^_geo_ would be expected based on the decreased availability of Ni sites, yet a charge transfer of only 0.012 ± 0.001 mC cm^−2^_geo_ is found. Therefore, there is a decreasing fraction of electrochemically active Ni sites upon increase in Co at.%. Furthermore, spinel films are characterized by a fixed onset potential over 500 CVs (Figure S6, Supporting Information). This points to a surface‐confined electrochemical activity for the cobalt‐rich spinel films, as opposed to the bulk activation observed for cobalt‐poor rock‐salt films.

**Figure 4:**
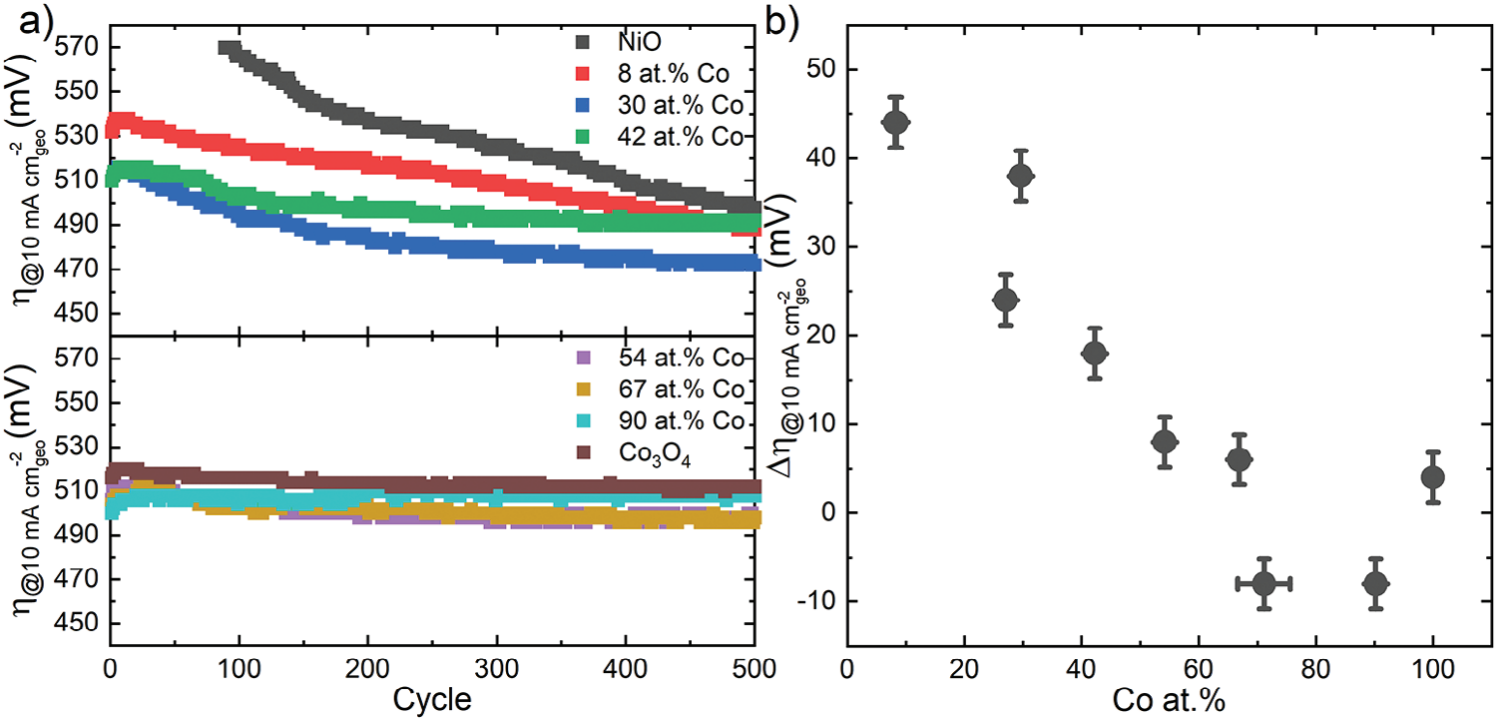
a) The evolution of the overpotential as a function of number of CV cycles for different Co at.% films. b) The change in overpotential, defined as the difference between the overpotential in cycle 1 with respect to cycle 500 such that a large positive value describes an extended activation, as a function of the Co at.%. Note that the overpotential is not defined for less than 100 CV cycles activated NiO, since the current density of 10 mA cm^−2^_geo_ was not reached below 1.8 V vs RHE.

The electrochemical performance and activation can be further analyzed by studying the overpotential required to reach a current of 10 mA cm^−2^_geo_, as a function of the number of CV cycles (**Figure** **4**). No overpotential is defined for the NiO films below ≈100 CV cycles, as the current density of 10 mA cm^−2^_geo_ was not reached within the cycling range of 1.2–1.8 V vs RHE. All films show similar overpotential ranges, independent of the initial composition or activation. This suggests that all films have a similar OER active phase, which is expected to be the (oxy)hydroxide phase.

A continuous activation is observed for the rock‐salt films (Figure 4a). The electrochemical activity of the NCO films might be attributed to the presence of electrolyte‐based iron impurities.^[^ ^18^, ^23^, ^70^, ^78^, ^79^, ^80^, ^81^, ^82^ ^]^ Even though high purity KOH is used for this study to control electrolyte impurities, trace amount of iron (≤6 at.% Fe) have been detected for the films with <30 at.% Co after electrochemical activation. No iron was observed for films with >30 at.% Co or for the pristine NCO films. The iron relative content is represented as a percentage of the total metal content in the film. Iron traces are present only at the surface of the film with similar concentrations per active site for various chemical compositions and activation cycles (for further elaboration see Page S8, Supporting Information). This indicates that, even though trace amounts of Fe promote the activity, the prolonged electrochemical activation observed in this work results from the modifications which the bulk of the electrocatalyst undergoes upon cycling. Additionally, similar iron impurity levels are expected to be inevitable for AEMWE applications, due to the other electrolyzer components.

Spinel films, on the other hand, show a constant overpotential over 500 CV cycles. The addition of nickel in the spinel structure slightly lowers the overpotential of these films as compared to Co_3_O_4_, with the 54 at.% Co and 67 at.% Co films showing similar low overpotentials of 500 mV. This can be attributed to the Ni^3+^ states in the inverse spinel, which has previously been shown to alter the electronic structure of Co_3_O_4_ to favor the adsorption of OH surface intermediates and lower the energy barrier for electron transfer to improve the OER kinetics.^[^ ^35^ ^]^

The activation is quantified in Figure 4b by the change in overpotential Δη, defined as the difference between the overpotential in cycle 1 with respect to cycle 500 such that a large positive value describes an extended activation upon decrease in overpotential, as a function of the Co at.%. The ability of rock‐salt films to activate, is observed to scale directly with the concentration of cobalt present in the film. After activation the 30 at.% Co rock‐salt film shows the best OER activity, at an overpotential of 470 mV. To further validate these results, a prolonged activation test is done on a Ni rich rock‐salt film (9 at.% Co) and a Co‐rich spinel film (94 at.% Co). The rock‐salt film shows a continuous activation over 1000 CV cycles, whilst the electrochemical activity of the spinel phase remains constant (Figure S7, Supporting Information).

### 2.3 Chemical Characterization After Electrochemical Activation

**Figure 5:**
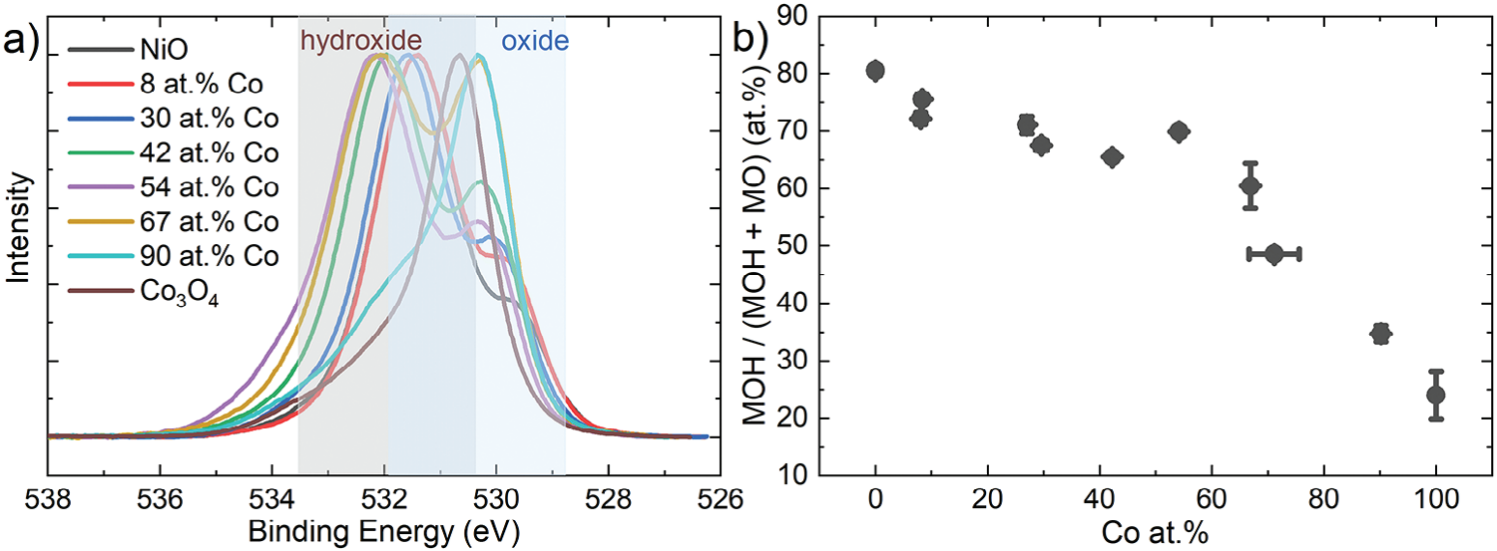
a) XPS of the O1s spectra of the films after 500 CV cycle activation, normalized to the maximum intensity of each trace. b) Ratio of hydroxide to (oxide + hydroxide) contributions derived from (a).

The changes in the chemical nature of the films after electrochemical cycling are analyzed by XPS in order to identify the nature of the electrochemical activation of the film (**Figure** **5**). First, no changes in the Co at.% are observed after CV cycling (Figure S8a‐e, Supporting Information), indicating that there is no leaching of either cobalt or nickel from the film.

The O1s spectra (Figure 5a), on the other hand, show significant changes after electrochemical activation. Films with a cobalt content below 65 at.% Co show a dominant feature located around 532 eV, whilst the feature located around 530 eV remains dominant for the 90 at.% Co and Co_3_O_4_ film. Careful analysis shows that the 532 eV feature is shifted to slightly lower binding energies as compared to the metal oxide O1s shoulder observed for the pristine films (Figure S8f, Supporting Information). Taking into account the oxidizing nature of OER, this feature is ascribed to the formation of a hydroxide phase.^[^ ^57^, ^58^, ^59^, ^60^ ^]^ Note that the metal oxide might still (partially) contribute to this hydroxide feature, such that only relative comparisons between films are possible. The fraction of metal hydroxide as compared to metal oxide is determined (Figure S3, Supporting Information) and visualized as a function of the Co at.% (Figure 5b). An almost linear decrease of hydroxide fraction is observed with increasing Co at.%. The hydroxide formation follows a similar trend to the change in overpotential discussed previously, suggesting that the change in overpotential directly correlates to hydroxide layer thickness (Figure S9, Supporting Information). This hypothesis is supported by angle‐resolved XPS measurements (Figure S10, Supporting Information) which shows an evenly distributed hydroxide layer throughout the 5–10 nm XPS probing depth of the 30 at.% Co rock‐salt film whilst the hydroxide phase is concentrated on only the surface of the 67 at.% Co spinel film. This further confirms that electrocatalytic activity of the spinel films is occurring at the surface, whilst rock‐salt films undergo bulk activation. Note that no quantitative information on the thickness of the surface layer can be inferred, due to the roughness of the FTO substrate.

Analysis of the Ni2p and Co2p XPS spectra (Page S13, Figure S11, Supporting Information) confirms the transition to predominantly nickel hydroxide for films <65 at.% Co. For higher cobalt concentrations, mainly Ni^3+^ states are present as an oxide. Mixed Co^2+^/Co^3+^ oxidation states are observed for all films, although rock‐salt films retain larger fractions of Co^2+^ remains, possibly in the form of Co(OH)_2_.^[^ ^40^, ^58^, ^59^, ^83^, ^84^ ^]^ Note that the bulk of the film still retains its oxide structure as observed by GIXRD (Figure S12, Supporting Information).

In summary, the hydroxide phase can be considered as fingerprint for the electrochemically active material. The bulk‐activation of rock‐salt films converts thicker oxide layers to hydroxide, whilst the surface‐limited electrochemical activity of the spinel films creates a thin surface hydroxide layer. The inclusion of cobalt in the films is observed to limit the bulk hydroxide conversion, probably as it partially converts to a more Co^3+^ rich phase.

The broad range of cobalt concentrations studied here, in combination with a prolonged activation process allow us to combine recent insights on the OER performance of Co_3_O_4_, CoO, and NiO into one coherent explanation for NCO films. The incorporation of cobalt in rock‐salt NCO enhances the conductivity (Figure S5, Supporting Information) such that an improved OER activity is observed. Additionally, the NCO films form a layered hydroxide structure when in contact with the electrolyte that reversibly transforms into oxyhydroxide during CV cycling. Water and anions can get intercalated in this layered structure during cycling,^[^ ^23^, ^71^, ^73^ ^]^ such that more bulk oxide can get activated to (oxy)hydroxide. This transformation is limited by the presence of cobalt, which partially converts to a more Co^3+^ rich phase, such that thinner hydroxide layers are formed. This was previously also observed for rock‐salt based cobalt oxide which converts to a mix of distorted Co_3_O_4_ and amorphous cobalt (oxy)hydroxide upon electrochemical activation.^[^ ^24^, ^29^ ^]^ These observations are also consistent with the higher sheet‐like morphology stability of cobalt hydroxide as compared to nickel hydroxide_._^[^ ^73^, ^85^, ^86^ ^]^ Films above 50 at.% Co are predominantly in spinel structure, where the hydroxide conversion and therefore the OER activity is limited to the surface. However, slightly thicker hydroxide layers are still observed for the 54 at.% Co and 67 at.% Co films, possibly due to the larger lattice parameter. The presence of additional Ni^3+^ oxidation states in 54 at.% Co and 67 at.% Co films alter the electronic structure of Co_3_O_4_ to favor the adsorption of OH surface intermediates and lower the energy barrier for electron transfer to improve the OER kinetics.^[^ ^35^ ^]^ All films possibly contain trace amounts of electrolyte originated iron on the surface, which facilitates the initial electrochemical activity but does not control the activation process.

### 2.4 Decoupling Intrinsic Activity and Electrochemical Surface Area

**Figure 6:**
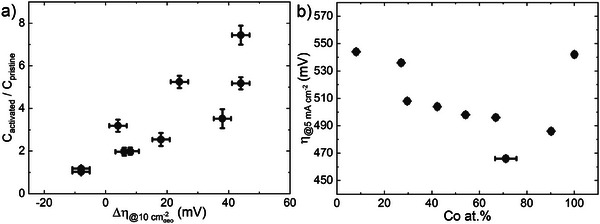
a) The change in ECSA, defined as the adsorbate capacitance of the activated film after 500 CV cycles (C_activated_) divided by the adsorbate capacitance of the pristine film (C_pristine_), as a function of the change in overpotential Δη = η_activated_−η_pristine_. b) The corrected overpotential at a current density of 5 mA cm^−2^ adapted to the change in ECSA as a function of the Co at.%.

The difference in electrochemical activation between Ni‐rich and Co‐rich NCO films can be the result of ECSA variations upon electrochemical testing (see Section 4.3; Page S14, Supporting Information). The variation in ECSA upon testing is described by the ratio in adsorbate capacitance between the pristine and activated films. This method assumes that the active materials remain the same during cycling. The correlation between the changes in ECSA of the deposited films and the activation of the films is visualized in **Figure** **6**a and shows an almost linear trend, suggesting that the activation process is dictated by an increase in active sites. This is highlighted by the 8 at.% Co rock‐salt film which shows a factor 7.5 ± 0.5 increase in active sites, whilst the number of active sites is constant for the 90 at.% Co spinel films (Figure S13, Supporting Information).

The change in ECSA is employed to normalize the current density to determine a corrected overpotential that reflects the intrinsic film activity (see Section 4.3). This normalization, however, lowers the current density to such an extent that the overpotential must be extracted at a current density of 5 mA cm^−2^ instead of 10 mA cm^−2^. The overpotential is elected as metric to facilitate comparison to previous measurements in this work as well as in literature. Note that the overpotentials corrected to ECSA are adopted to compare among films instead of providing absolute values. This method furthermore does not correct for the number of active sites per geometric surface area, as they are considered intrinsic material properties of the rock‐salt and spinel structures. The validity of the approach is confirmed by a comparison to the more traditionally employed turnover frequencies (see Page S15, Figures S14 and S15, Supporting Information).

Interestingly, these corrected overpotentials (Figure 6b) show an opposite activity trend as compared to those values shown in Figure 4, yet closely resemble the trend of electrocatalytic activity in the first CV cycle. All spinel NCO films outperform Co_3_O_4_ and all rock‐salt NCO films outperform NiO, validating that there is a synergistic effect between cobalt and nickel. This synergistic effect is possibly related to the enhanced conductivity observed for the mixed oxides. The spinel oxide films furthermore outperform the rock‐salt based films, with an optimal performance for the 71 at.% Co spinel oxide film. The observed optimal performance of the 30 at.% Co film before correction therefore indicates that the rock‐salt activity originates from the increase in active site density upon increase in ECSA, as result of the bulk formation of hydroxide species. These findings can be further validated by the change in ECSA of the 1000 cycle activated films. A significant change in ECSA is observed for the 9 at.% Co film (C_activated_/C_pristine_ = 11 ± 2), whilst the 94 at.% Co film remains almost constant (C_activated_/C_pristine_ = 1.4 ± 0.1). The corrected current densities of these films (Figure S16, Supporting Information) fall within the trend observed for the 500 CV cycle activated films, further validating the chosen approach, and confirming the attribution of activation to the increase in active sites.

## 3 Conclusion

In this study, we have adopted a thin film model system by ALD to unravel the structure – OER activity relation of NCO films. Extensive film characterization, prior to electrochemical activation, shows the transition of NCO from rock‐salt to spinel structure as a function of Co at.%. Electrochemical analysis discloses a synergistic effect between cobalt and nickel such that NCO films are more OER‐active than Co_3_O_4_ and NiO. All films show a similar overpotential range, which indicates a similar active phase: the formation of (oxy)hydroxide phase is confirmed to drive the OER activity of the films. The OER performance is further monitored over 500 CV cycles. Rock‐salt films are observed to continuously form a hydroxide phase during CV cycling, resulting in prolonged activation and an optimal OER‐activity for the 30 at.% Co after 500 cycles. The activation process proceeds in parallel with an increase in ECSA up to a factor 8 for rock‐salt films. Instead, the presence of cobalt limits the bulk hydroxide formation, such that a constant performance is observed for spinel type films over 500 cycles. Through an original approach we correct the overpotential for the change in ECSA which the NCO films undergo upon activation, and we conclude that Ni‐rich spinel films are intrinsically the most OER active.

In conclusion, we demonstrate that NCO films witness a dual electrochemical behavior, depending on their chemical composition: the stable surface‐active Co‐rich spinel type films and the dynamic bulk‐activated Ni‐rich rock‐salt films. When placing our results in perspective, we infer that working with thin films favors the adoption of rock‐salt NCO films due to their extensive activation process. High surface area 3D electrodes, on the other hand, would promote the application of spinel NCO films as the intrinsic activity is larger than the rock‐salt phase, whereas the high surface area provides a higher active site density. These observations are crucial for future implementation of the catalysts, as they indicate a system dependent optimal NCO composition. We can also conclude that rock‐salt NCOs are a sustainable alternative to the more commonly employed spinel films. Even though longer activation protocols are required to maximize their OER performance, sustainability is achieved by minimizing the Co at.% presence in the catalyst.^[^ ^43^ ^]^ Finally, these results emphasize the importance of prolonged electrochemical tests in combination with extensive material characterization techniques of well‐defined model system to study the activity and activation of electrocatalysts.

## 4 Experimental Section

### ALD Process

NCOs were deposited by adopting a home‐built plasma‐enhanced ALD reactor, described elsewhere.^[^ ^87^ ^]^ The system was equipped with an inductively coupled plasma source with a power supply operating at 13.56 MHz The pumping system comprises of a turbomolecular pump connected to a rotary vane pump, capable of reaching a base pressure below 1 × 10^−6^ mbar. During deposition, the substrate holder, suitable for fitting a 4 inch wafer, was heated to 300 °C whilst the reactor walls maintain a temperature of 100 °C. Cobaltocene (CoCp_2_, 98% purity, ACROS Organics) was heated to 80 °C and dosed using Ar carrier gas through a 100 °C line. The stainless steel cylindrical bubbler containing 1,1′‐dimethylnickelocene (Ni(^Me^Cp)_2_, 97% purity, Sigma–Aldrich) was heated to 55 °C and carried to the chamber using a 75 °C and Ar gas. Both precursors use a 100 W O_2_ plasma as co‐reactant.

**Scheme 1:**
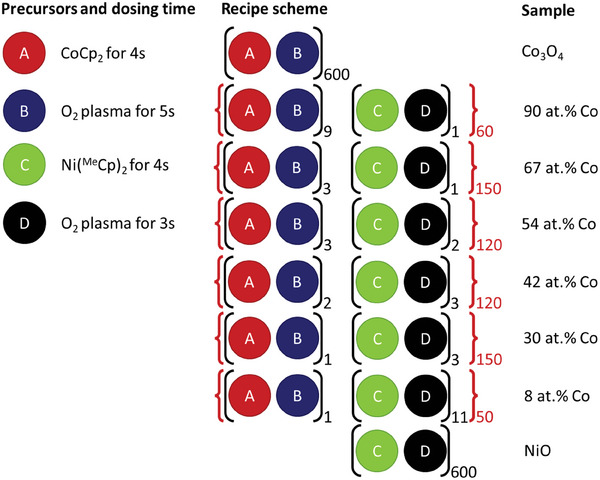
Schematic illustration of the ALD process employed for the NCO films. The precursor dosing is followed by an Ar purge/pump of 5s/1s, whilst the plasma dosing is followed by an O_2_ purge/pump of 1s/3s.

The films were prepared using the supercycle‐approach previously published.^[^ ^40^ ^]^ In short, the process of precursor dosing/Ar purge/pump of 4s/4s/5s for both ALD cycles in the supercycle was followed by O_2_ plasma exposure/O_2_ purging/pumping of 3s/1s/3s for the NiO_x_ half cycle and of 5s/1s/3s for the CoO_x_ half cycle. The cobalt atomic concentration was varied by changing the ratio of NiO_x_:CoO_x_ cycles within one supercycle (**Scheme** **1**). All samples were deposited by 600 ALD cycles with varying Co:Ni nominal ratios, resulting in a thickness in the range from 20 nm for NiO up to 35 nm for Co_3_O_4_ depending on the composition as determined by spectroscopic ellipsometry measurements on reference c‐Si wafers. The effective cobalt‐to‐nickel ratios, referred to as cobalt atomic concentrations (at.% Co) were determined by XPS analysis as at.% Co = 100%∙ Co / (Co + Ni). All films were deposited on 1.1 mm fluorine‐doped tin oxide (FTO) glass substrates obtained from redox.me, unless indicated otherwise. The substrates were cleaned for 5 min of subsequent soap water, acetone and IPA sonication and treated with a 15 min oxygen plasma directly before film deposition. Films were rinsed using demi‐water directly after electrochemical characterization.

### ALD Film Characterization

The crystal phases of the films were investigated by grazing incidence XRD at 0.4° offset using a Bruker Discover D8, utilizing Cu Κα (*λ* = 1.54060 Å) radiation in the 2*θ* range from 15° to 80° with 0.025° steps at a scan speed of 20 s per step. To minimize fluorescence effects between Co and the Cu source,^[^ ^88^ ^]^ the detector energy discriminator was raised to 7000 eV. All measurements were internally calibrated to the (110) SnO_2_ feature at 26.6°. XPS was performed to investigate the chemical composition of the films using a Themo Scientific KA1066 spectrometer equipped with monochromatic AL Κα x‐rays. The binding axis was calibrated by using the adventitious C1s peak at 248.8 eV as a reference. Measurements were taken at five positions on the film, and the error in Co at.% was determined using the standard deviation. Note that the oxygen content in the film cannot be accurately determined through comparison of the 3p region with the O1s region.^[^ ^89^ ^]^ Angle‐resolved measurements were obtained using a specialized tilt sample holder (see Page S12, Supporting Information). The at.% Co was verified using previously published^[^ ^40^ ^]^ particle‐induced x‐ray emission (PIXE) spectra on films deposited on Si. Note that PIXE was only employed for relative cobalt content determination, as opposed to the Rutherford Backscattering used in previous work for quantitative analysis. For PIXE, a 2.7 MeV H^+^ beam at perpendicular incidence has been applied. The x‐ray detector was positioned at an angle of 45° between the beam and the specimen normal, and 30 µm Kapton has been used as an absorber. The spectra have been analyzed with the Gupix^[^ ^90^ ^]^ PIXE simulation package.

The root mean square (RMS) roughness was determined by AFM using a Bruker Dimension Icon in PeakForce tapping mode in combination with a ScanAsyst‐Air tip. Values were obtained by the average value and standard deviation of the RMS roughness as determined using the NanoScope Analysis 2.0 software on five 0.5 × 0.5 µm locations on each film. The surface morphology was further investigated using a Zeiss Sigma field‐emission scanning electron microscope. A Signatone four‐point probe in combination with a Keithley 2300 Sourcemeter was employed to measure the electrical resistivity at room temperature. Highly resistive 450 nm SiO_2_‐coated c‐Si substrates were used for conductivity measurements.

### Electrochemical Characterization

The electrochemical OER performance was measured in a standard three‐electrode cell configuration connected to a potentiostat (Ivium Technologies). The working anode ALD film was assembled in a 25 × 25 mm sample holder with an aperture that allows 1 cm^2^ geometric area to be exposed to the electrolyte. A graphite rod (6/70 mm, redox.me) was used as a counter electrode, in combination with a reversible hydrogen electrode (RHE) reference electrode^[^ ^91^ ^]^ (mini HydroFlex, Gaskatel). Measurements were executed in 90 mL of 1 m KOH (99.98% trace metal basis, Fisher Scientific) aqueous solution prepared from pellets, with 40 ± 10 ppb Fe as obtained with inductively coupled plasma‐mass‐spectroscopy (ICP‐MS). Electrochemical impedance spectroscopy (EIS) was used to determine the cell resistance, and 80% iR compensation was applied to the measurements. The electrochemical techniques consist of 1) linear sweep voltammetry (LSV) at 1 mV s^−1^, 2) EIS at 1.6 V vs RHE, 3) two cyclic voltammetry (CV) cycles at 20 mV s^−1^, 4) 500 CV cycles at 50 mV s^−1^, 5) LSV at 1 mV s^−1^, 6) EIS at 1.6 V vs RHE and 7) two CV cycles at 20 mV s^−1^. A measurement range of 1.2–1.8 V vs RHE was employed for all potentiodynamic measurements. Data analysis regarding the redox features was based on measurements (3), (5) and (7), whilst the activation behavior was studied using measurement (5). The integrated charge Q of the oxidation wave was determined through integration of the redox feature between 1.25 and 1.50 V vs RHE with a linear background. The current densities were based on 1 cm^2^ geometric surface area, indicated as mA cm^−2^_geo_.

The change in ECSA was determined using the adsorbate capacitance of the activated film after 500 CV cycles (C_activated_) divided by the adsorbate capacitance of the pristine film (C_pristine_). The adsorbate capacitance was determined by fitting the equivalent electrical circuit presented by Watzele et al.^[^ ^92^ ^]^ to EIS measurements (2) and (6) of the electrochemical techniques (see Figures S17–S19, Tables S2 and S3, Supporting Information). A corrected overpotential that reflects the intrinsic film activity was determined from the corrected current density, which was calculated as the current density at cycle 500 divided by (C_activated_ /C_fresh_).

## Conflict of Interest

The authors declare no conflict of interest.

## Supporting information

Supporting Information

## Data Availability

The data that support the findings of this study are openly available in [Zenodo] at [10.5281/zenodo.10371385]
