# Supplementary material for: Unraveling the Role of the Stoichiometry of Atomic Layer Deposited Nickel Cobalt Oxides on the Oxygen Evolution Reaction
Source: Adv Sci (Weinh). 2024 Jul 3;11(32):2405188. doi: 10.1002/advs.202405188 (PMC11348001; doi:10.1002/advs.202405188)
Supplement: Supplementary file 1 — Supporting Information [file ADVS-11-2405188-s001.pdf]

ADVANCED SCIENCE
Open Access

Supporting Information

for *Adv. Sci.*, DOI 10.1002/advs.202405188

Unraveling the Role of the Stoichiometry of Atomic Layer Deposited Nickel Cobalt Oxides on the Oxygen Evolution Reaction

Renée T. M. van Limpt, Mengmeng Lao, Mihalis N. Tsampas and Mariadriana Creatore**

Supporting Information

Unraveling the Role of the Stoichiometry of ALD Nickel Cobalt Oxides on the Oxygen Evolution Reaction

Renee T.M. van Limpt , Mengmeng Lao, Mihalis N. Tsampas and Mariadriana Creatore

ALD films characterization

**Composition**	**Root mean square (RMS) roughness**
FTO glass	11±2 nm
NiO	11±2 nm
8 at.% Co	11±2 nm
27 at.% Co	13±3 nm
42 at.% Co	10±1 nm
54 at.% Co	11±1 nm
67 at.% Co	9±1 nm
90 at.% Co	9±1 nm
Co_3O_4	10±2 nm

Table S1 Root mean square (RMS) roughness determined by AFM on 0.5·0.5 μm^2 locations on the FTO substrate and pristine films.

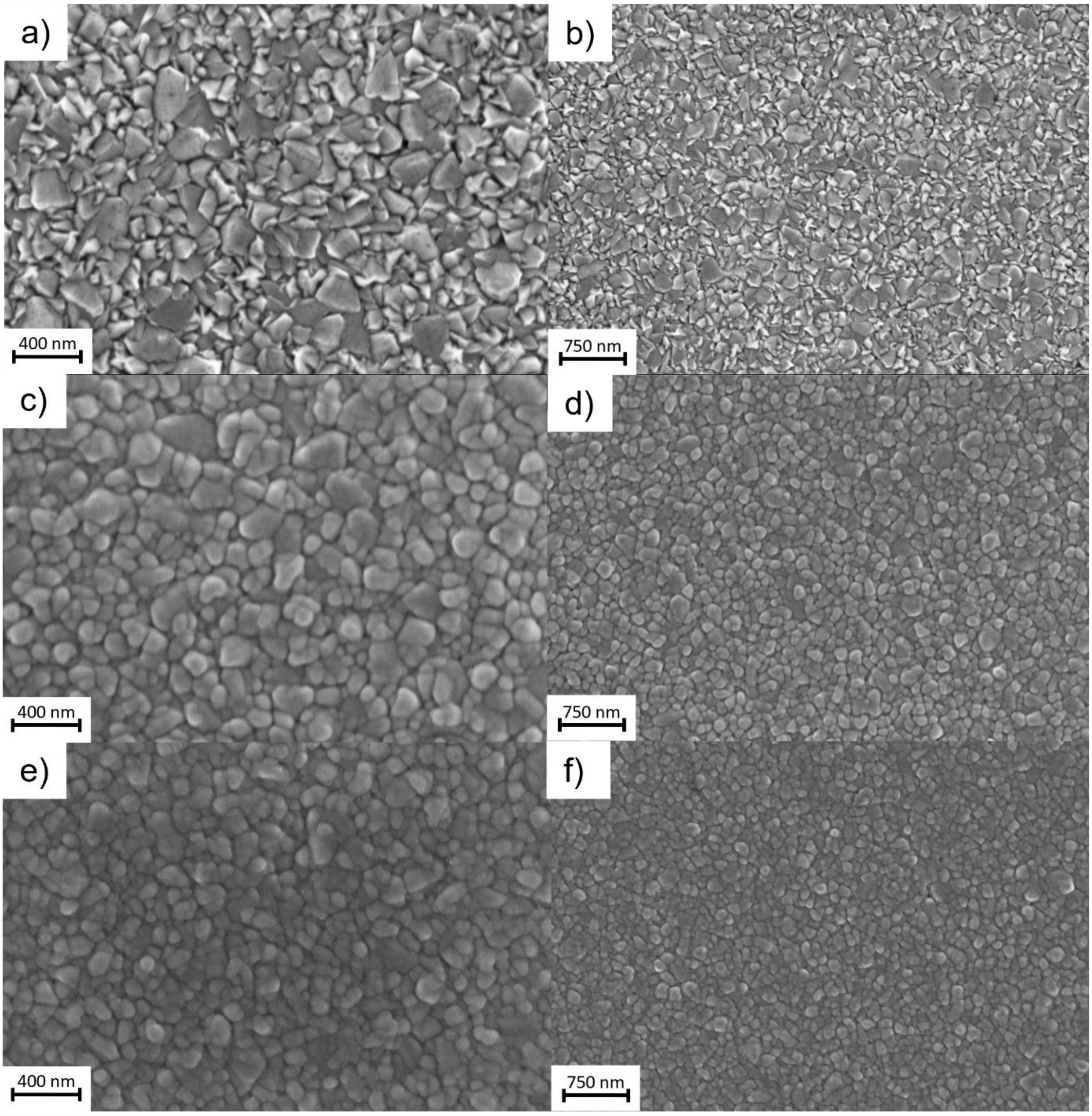

Figure S1 Scanning electron microscopy (SEM) image of (a,b) FTO glass, (c,d) 8 at.% Co and (e, f) 90 at.% Co films deposited on FTO.

XPS Analysis

The chemical composition and oxidation states of the films are investigated using X-ray Photoelectron Spectroscopy (XPS) (**Figure 1, 5, S2, S3, S8-11**). The exact assignment of specific chemical environments in the first-row transition metal oxides is non-trivial due to the complexity of the spectra and the overlap in binding energies of features of various transition metal oxides.[1–6] The presence of Auger features of cobalt (hydr)oxide in the Ni2p region and of nickel (hydr)oxide in the Co2p region[7] make chemical composition determination reliant on the (overlapping) Co3p and Ni3p spectra.

The chemical composition is expressed as the at.% Co =100% · Co / (Co + Ni) and is extracted using the Ni3p and Co3p spectra (**Figure S2**). Each individual oxide is fitted with 4 mathematical peaks, such that a stable and accurate fit is obtained. Slight variation in the binding energy of the primary fitted peak is allowed to account for different 3p spectra of oxides and (oxy)hydroxides such that the same fitting procedure can be employed before and after electrochemical activation.[5] The mixed oxides are fitted with a combination of the fits obtained for a Co_3O_4 and NiO reference spectra. For iron incorporation, the same procedure is applied based on a FeO_x reference film.

Interpretation of the Ni2p and Co2p spectrum requires knowledge of the oxidation state of the film; specifically, whether the film is (partially) a hydroxide or a pure oxide. This can be deduced from the O1s spectrum (**Figure S2**). Due to the surface sensitive nature of XPS, generally at least two features are generated: one for bulk or lattice oxygen and one for surface bound oxygen. The feature assigned to the lattice oxygen shows a binding energy of ~530 eV with a small shift of ~0.6 eV towards higher binding energies for cobalt oxide as compared to nickel oxide. Surface bound oxygen, in the form of absorbed water of organics is typically assigned to features at 533 eV. Yet Frankcombe *et al.*[8] have recently demonstrated that the often observed feature at ~532 eV should also be ascribed to surface chemisorbed water or adsorbed hydrogen passivated surface oxygen. Note that this observation does not contradict the assignment of the 533 eV feature, as this feature originates from more loosely bound water. The feature at ~532 eV can, however, also be assigned to the formation of a hydroxide phase. Careful analysis of this shoulder by Biesinger *et al.*[3] shows that this feature is observed at slightly higher binding energies for adsorbed water than for hydroxides. In summary, the oxides will show a main feature at ~530 eV and a shoulder around ~532 eV due to adsorbed water. Pure hydroxide films, on the other hand, will only show a feature at ~532 eV. Note that it is difficult to distinguish the nature of the ~532 eV feature, yet comparison between different films (e.g. before and after electrochemical activation) and with different elemental detection techniques (e.g. elastic recoil detection) provides hints.[1–3,5]

The chemical environment of the Ni species can be determined mainly from the 850-860 eV region of the Ni2p spectrum. The feature at 854 eV is ascribed to the Ni^{2+} oxidation state, whilst the feature at 856 eV can be ascribed to both the Ni^{3+} and the Ni^{2+} oxidation state. The nature of the film, whether it forms an oxide or a hydroxide, is crucial for interpretation of the 856 eV feature. If the film is in a hydroxide phase, therefore consisting of Ni^{2+}, the Ni2p spectrum will display a feature at 856 eV without the presence of a feature at 854 eV. If the deposited material forms an oxide, the feature in 856 eV is expected to partially originate from Ni^{3+} states, where an increase in the shoulder is indicative of more Ni^{3+} states. The presence of a feature at 856 eV does therefore not necessarily indicate the presence of Ni^{3+} states. Note that an oxide will

always also show some feature at 854 eV, albeit high concentrations of Ni^{3+} might result in coalescence or dominance of the 856 eV 'shoulder'. The proportionality between both features can, nonetheless, be employed as a descriptor for the Ni^{3+}-to-Ni^{2+} ratio.[1,2,5–7]

The chemical environment of the Co species is typically determined from the satellite features between 780-795 eV in the Co2p spectrum. Chemical state identification following this route is, however, limited in nickel cobalt oxide (NCO) thin films due to the presence of an NiO_x Auger feature directly blow the $Co2p^{1/2}$ feature. Two alternative characteristics of the Co2p spectrum can be employed for chemical state identification. An energy split of 15 eV between the $2p^{1/2}$ and $2p^{3/2}$ features is indicative of the mixed Co^{2+}/Co^{3+} features of the Co_3O_4 structure, whilst a larger split of 16 eV is indicative of the Co^{2+} oxidation state observed in CoO. These observations can furthermore be confirmed by the satellite feature between 800-810 eV, where a more intense satellite feature at slightly lower binding energy is indicative of a lower Co^{3+}-to-Co^{2+} ratio. The Co2p spectra for the Co^{2+} based oxide and hydroxide are very similar, highlighting again the importance of proper interpretation of the O1s spectrum.[3–7] Also note that for both interpretation of the Co2p and Ni2p spectrum an adequate background should be selected. It is recommended to fit the background over a wide range with possibly a linear correction (760-810 eV for Co2p and 845-890 eV for Ni2p) to assure proper determination of the satellite features.[2,4]

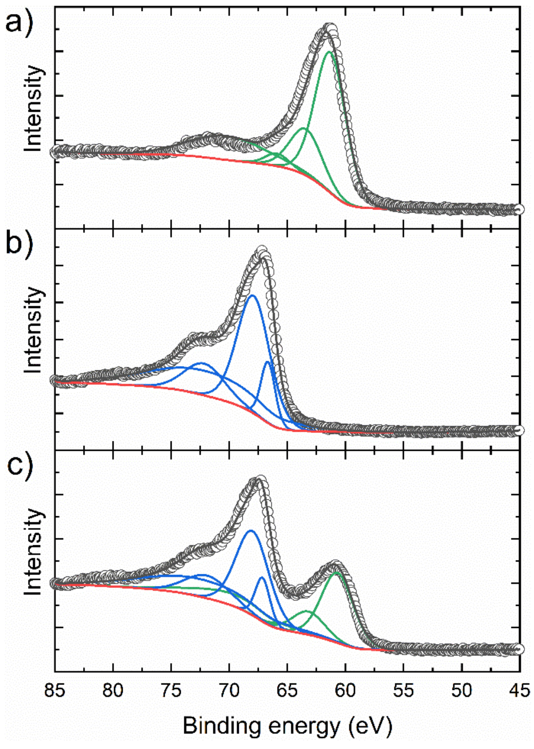

Figure S2 Fitting procedure for the overlapping Ni3p and Co3p regions of (a) Co_3O_4, (b) NiO and (c) NCO (42 at.% Co) used to determine the cobalt concentration as a function of the total metal content. All figures share the x-axis scale. Note that a purely mathematical fitting procedure is employed to fit the features.

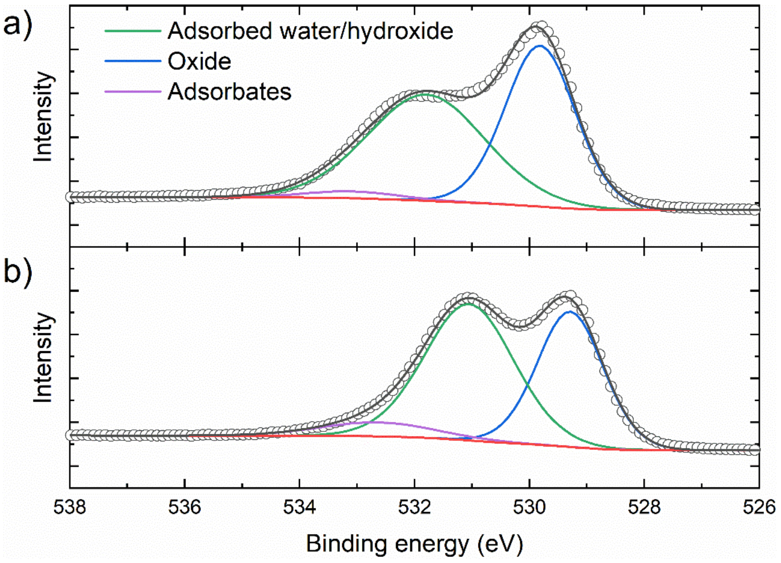

Figure S3 Fitting procedure for the O1s feature, illustrated by the spectrum (a) before and (b) after electrochemical activation of a 67 at.% Co film. The figures share the x-axis scale and legend.

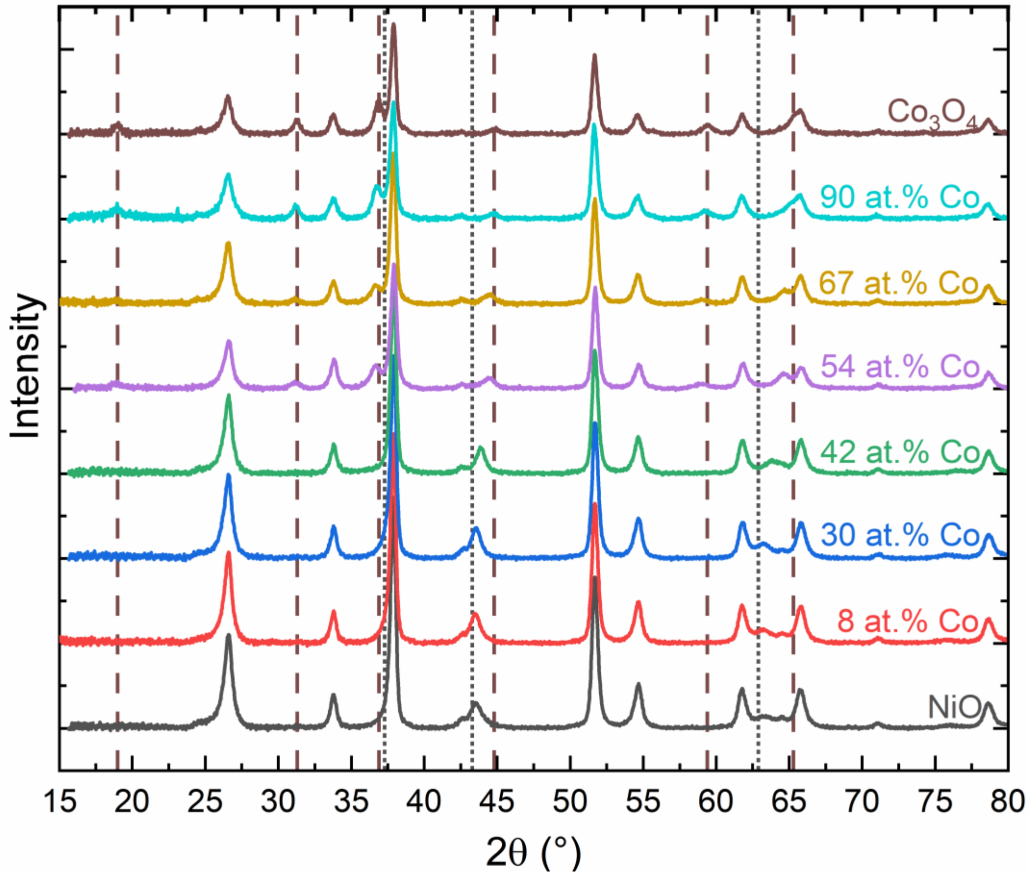

Figure S4 Grazing incidence x-ray diffraction (GIXRD) measurements of the various NCO films. The feature positions of Co_3O_4 (brown, dashed line) and NiO (black, dotted line) as obtained from the ICSD database (data cards 36256 and 9866 respectively) are indicated by vertical guides to the eye.

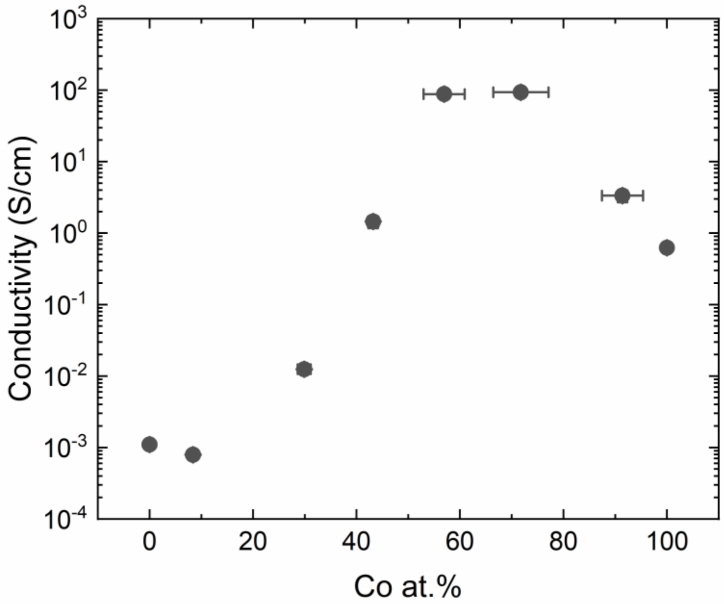

Figure S5 Four-point probe conductivity measurements of NCO films.

Electrochemical characterization

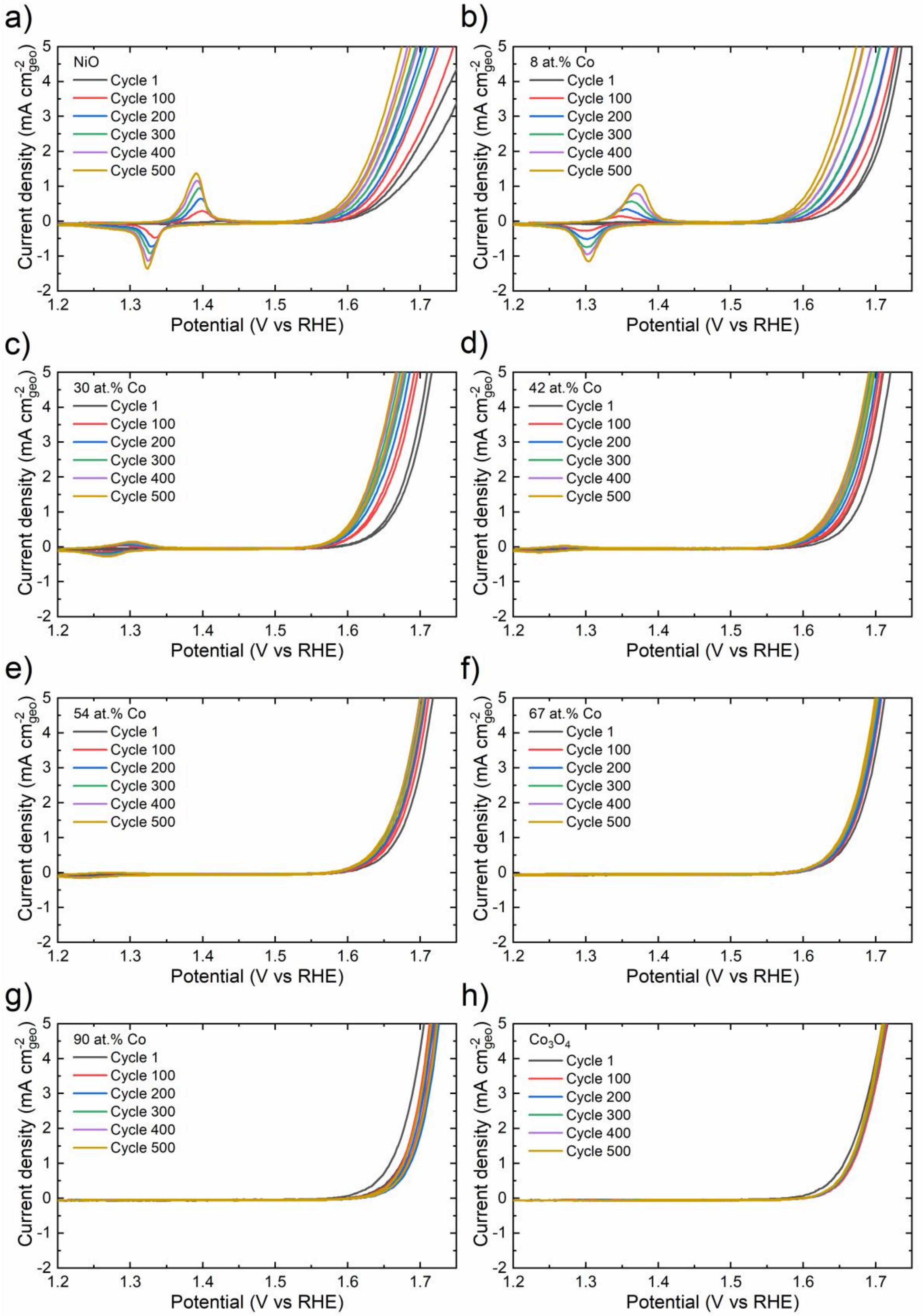

Figure S6 Electrochemical measurements during 500 cyclic voltammetry (CV) cycles in 1M ultrapure KOH for NCO films with various Co at.%.

Iron incorporation

Nickel is known to scavenge iron from the KOH solution, and the presence of iron is reported to enhance the activity of (cobalt) nickel oxide films.[9–16] Even though high purity KOH is used for this study, trace amounts of iron have already shown to enhance the OER activity. XPS studies after 500 CV cycles of electrochemical activation show that Fe is present in selected films; 6±2 at% Fe in NiO, 3±1 at.% Fe in the 8 at.% Co film and 2.8±0.6 at.% Fe in the 30 at.% Co film. Note that the presence could only be detected using the 3p spectra, as the Fe2p spectrum shows a significant overlap with Auger features from nickel oxide and cobalt oxide. The iron relative content is represented as a percentage of the total metal content in the film. No iron is detected for films with higher cobalt concentration.

The redox feature of the films does not shift to higher potentials upon cycling (**Figure S6**), indicating that Fe is not present in the bulk of the film but only at the surface.[9,15,17] Surface incorporation of Fe is known to occur rapidly once the film is subjected to the electrolyte.[9,15-18] The at.% Fe is normalized to the change in electrochemical surface area (ECSA) to obtain the amount of iron per active (surface) site. This results in 0.5±0.2 at.% Fe for the 8 at.% Co and 0.6±0.2 at.% Fe for the 30 at.% Co film. These values are below the typical detection limit for XPS, validating why no iron is observed for higher at.% Co films where less material is activated.

Prolonged activation tests are carried out on a Ni rich rock-salt film (9 at.% Co) and a Co-rich spinel film (94 at.% Co) to further investigate the presence of iron (**Figure S7, S16**). After electrochemical testing for 1000 cycles, 11±2 at.% Fe is observed for the 9 at.% Co film whilst no Fe is observed for the 94 at.% Co film. Normalization of this value to the variation in electrochemical surface area results in an iron contamination of 0.5±0.1 at.% Fe. This equals the ECSA normalized iron film contamination of 0.5±0.2 at.% Fe of the 500 cycle activated 8 at.% Co film. Hence, this shows that an equal amount of iron is present per active surface site, highlighting that the activation process does not result from additional iron incorporation. Therefore, it is safe to conclude that, even though trace amount of Fe stimulates the OER activity, the prolonged electrochemical activation observed in this work results from the modifications which the bulk of the electrocatalyst undergoes upon cycling.

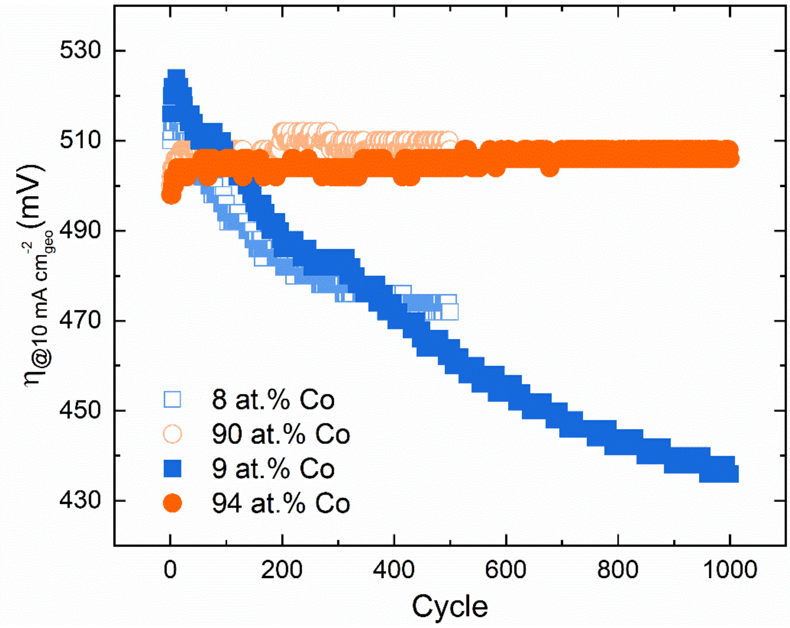

Figure S7 Overpotential, defined as the potential required to reach 10 mA cm^{-2}_{geo} as a function of number of CV cycles for films activated for 1000 cycles (filled data points) and 500 cycles (open data points).

Film characterization after electrochemical testing

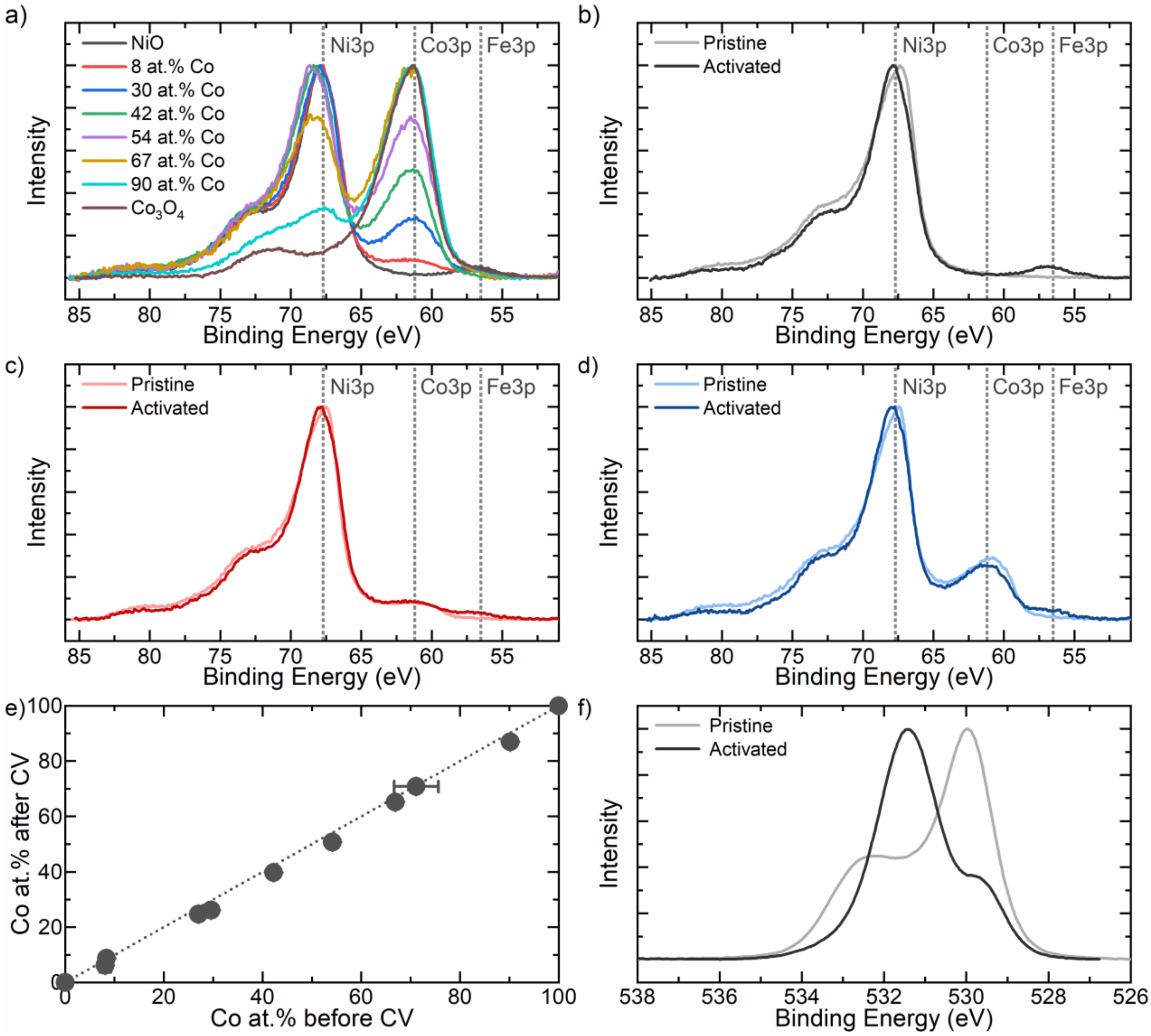

Figure S8 Additional chemical characterization of the films after electrochemical activation. (a) The 3p spectra normalized to the maximum of each trace. The 3p spectra of (b) NiO, (c) 8 at.% Co and (d) 30 at.% Co of pristine and activated films are included to highlight the Fe incorporation. The vertical lines are added as a guide to the eye. (e) Co at.% extracted from the 3p spectra of pristine and activated films. The dotted line represents a stable Co at.% prior and after cyclic voltammetry and is added as a guide to the eye. (f) The change in O1s spectrum before and after electrochemical activation visualized for NiO.

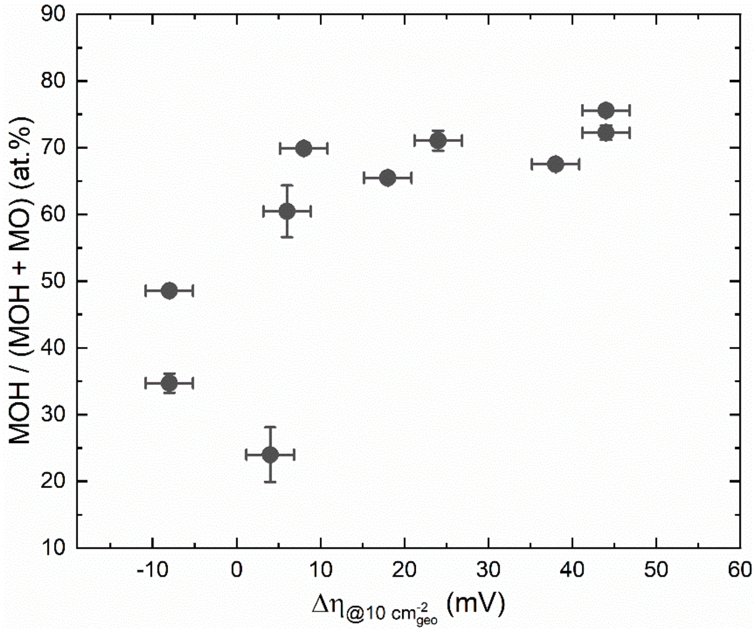

Figure S9 The fraction of hydroxide phase after electrochemical testing as a function of the change in overpotential, defined as the difference between the overpotential in cycle 1 with respect to cycle 500 such that a large positive value describes an extended activation.

Angle-resolved x-ray photoelectron spectroscopy

Angle-resolved x-ray photoelectron spectroscopy (ARXPS) can provide additional information on the distribution of the film composition along the depth of the film. Increment in the x-ray detection angle with regard to the surface normal makes the technique more surface sensitive, without the intermixing and preferential sputtering observed in the case of a depth profile by Ar sputtering.

Measurements on an activated 30 at.% Co and a 67 at.% Co (**Figure S10**) film provide insight into the thickness of the hydroxide layer. The 30 at.% Co film shows an approximately stable metal hydroxide dominated spectrum, indicating that the whole surface region as probed by XPS is hydroxyl-group terminated. The 67 at.% Co film, on the other hand, initially shows an almost equal distribution of metal oxide and hydroxide phase. The hydroxide-to-oxide ratio is shown to start increasing for more surface sensitive angles indicating that a thinner surface hydroxide layer is present. Note that no quantitative information on the thickness of the surface layer can be inferred due to the roughness of the FTO substrate.

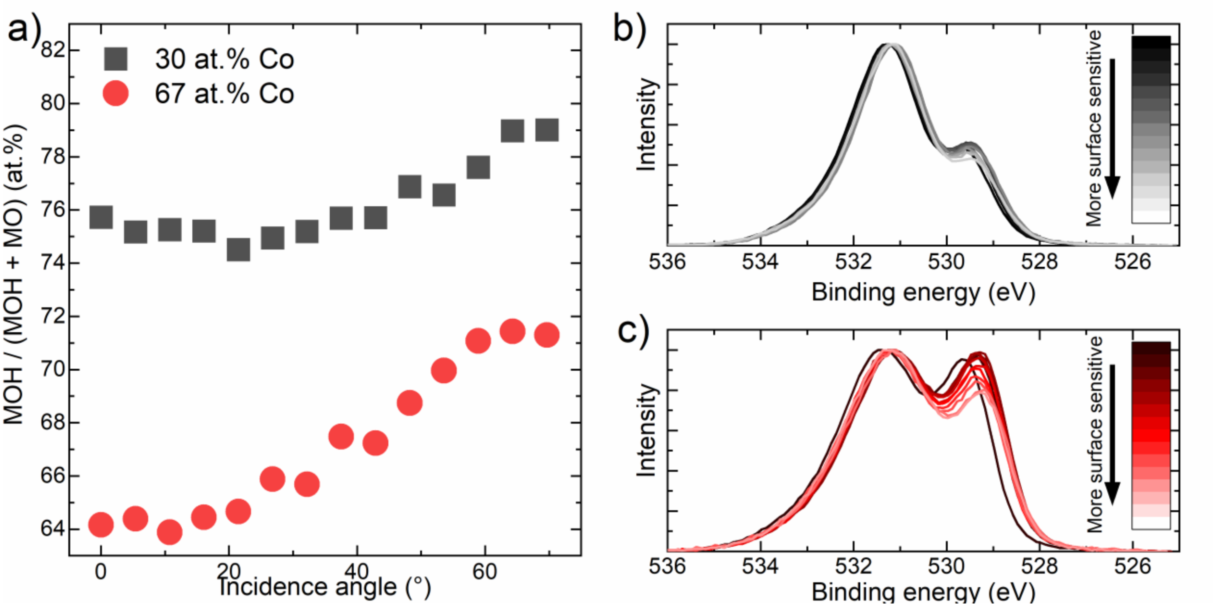

Figure S10 Additional chemical characterization of the films after electrochemical activation. (a) The percentage of hydroxide bonds (MOH) with regards to the total hydroxide and oxide (MOH + MO) bonds as a function of detection angle with regard to the surface normal as obtained from angle resolved x-ray photoelectron spectra. The individual spectra normalized to the trace maximum are visualized in figure (b) (30 at.% Co) and (c) (67 at.% Co). Increasing the detection angle with regard to the surface normal results in more surface sensitive measurements as indicated by the lighter trace color in the spectra. Both legends correspond to the same increment in incidence angle.

Analysis of the Ni2p spectra after electrochemical activation (**Figure S11a**) shows a dominant feature at 856 eV for all films. The 856 eV feature is attributed to the formation of nickel hydroxide for films <65 at.% Co, in line with the observation of the hydroxide dominated O1s spectrum. The 856 eV feature is assigned to mainly Ni^{3+} states with a possible small hydroxide Ni^{2+} contribution for cobalt concentrations >65 at.% Co, due to the dominance of the oxide feature in the O1s spectra. For all films a small contribution of the 854 eV feature is still present, with a larger contribution for higher Co at.% films. This contribution suggests that some Ni^{2+} based nickel oxide is still present.

The Co2p spectra (**Figure S11b**) show a decrease of the feature between 800-810 eV for low Co at.% as compared to the pristine films, such that similar traces are observed for all cobalt concentrations. The decreased feature indicates a mixed Co^{2+}/Co^{3+} oxidation state-based film.[3,5,6,19] However, a larger energy split of ~16 eV is still observed for the rock-salt films, indicating that some Co^{2+} remains, possibly in the form of $Co(OH)_2$ as derived from the hydroxide dominated O1s spectra.[3-7]

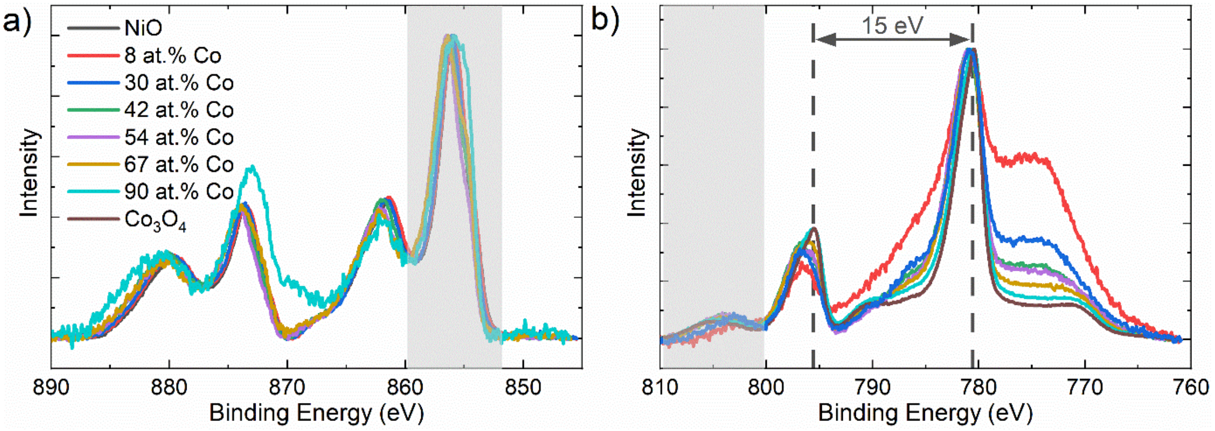

Figure S11 XPS of the (a) Ni2p and (d) Co2p spectra normalized to the maximum intensity of each trace. The legend presented in figure (a) is also valid for figure (b).

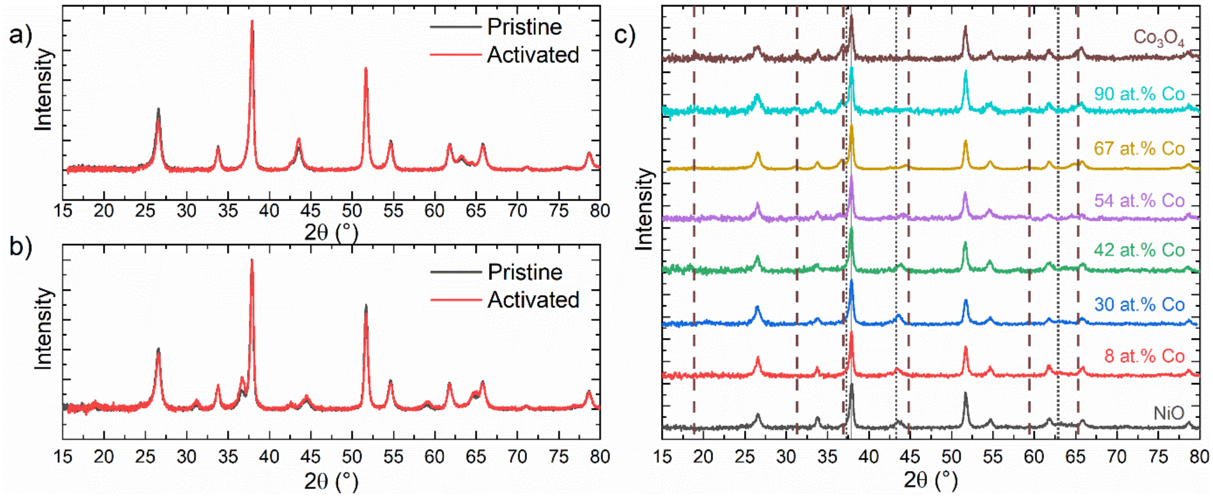

Figure S12 Grazing-incidence XRD of the (a) 30 at.% Co and (b) 67 at.% Co films before and after electrochemical testing and (c) all films after electrochemical testing with 500 CV cycles obtained at a scan rate of 1 s/step .

Decoupling intrinsic activity and ECSA

The change in ECSA is determined using the adsorbate capacitance (C_a), although conventionally, the ECSA is estimated using the double layer capacitance at non faradaic potentials.[20-23] However, Ni-based (hydr)oxides are known to be non-conductive before the Ni^{2+} to Ni^{3+} oxidation which makes capacitance measurements in this region less reliable, whilst measurements done at higher potential than the redox feature might be influenced by the oxygen evolution reaction (OER) activity.[21,24,25] Recent studies[21,24,26] have shown that it is more appropriate to use the adsorbate capacitance as derived from electrochemical impedance spectroscopy (EIS) for nickel (hydr)oxide based materials. The adsorbate capacitance originates from the OER intermediates at low overpotential[20,24,26], and is dependent on the catalyst material. The C_a is determined by fitting the equivalent electrical circuit presented by Watzele *et al.*[26] to an EIS measurement at 1.6 V vs RHE (**Figure S17-19, Table S2-3)**. The equivalent circuit is a combination of the double layer at the electrode and the reactant ad- and desorption and consists of an uncompensated resistance in series with a parallel connection of the double layer capacitance and the charge transfer resistance connected in series to a parallel adsorption RC element. Both Jeon *et al.*[24] and Watzele *et al.*[26] furthermore verified the validity of the circuit for various electrocatalyst systems on glassy carbon and inert supports. Typically, the adsorbate capacitance is normalized with respect to the specific adsorbate capacitance to determine the ECSA. The specific adsorbate capacitance, however, varies for different compositions of NCOs.[26] This study therefore focuses on the changes in the ECSA, defined as the adsorbate capacitance of the activated film after 500 CV cycles ($C_{activated}$) divided by the adsorbate capacitance of the pristine film ($C_{pristine}$). To fairly compare changes in ECSA, it is assumed that the (oxy)hydroxide is the active material for all CV cycles.

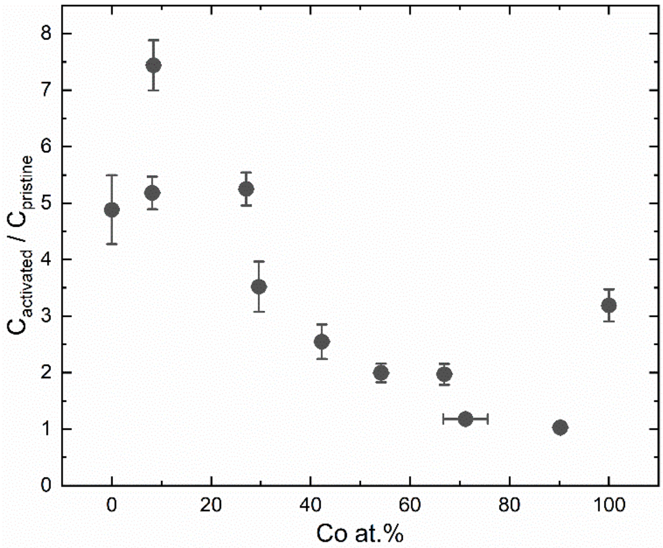

Figure S13 The change in electrochemical surface area as represented by the adsorbate capacitance ratio for activated and pristine films as a function of the cobalt at.%.

Apparent turnover frequency

The validity of the normalized overpotential approach is confirmed by comparison to the calculated turnover frequency (TOF). Calculation of the TOF requires the (surface) concentration of active sites, which is not easily derived for NCO films.[27] Hence, the calculation is based on the total number of Ni sites in a NiO film derived previously using Rutherford backscattering spectrometry[7]. For the NCO films, is assumed that all films have a similar total amount of metal sites as the amount of metal sites in the NiO film, and therefore a similar active surface concentration before activation, such that an apparent TOF (aTOF) can be calculated. The assumption is guided by the same number of ALD cycles in the process, and validated by the comparable densities of NiO and Co_3O_4 at 6.7±0.3 g/cm^3 and 6.2±0.3 g/cm^3 respectively.[7] In the following graphs we have used two normalization approaches; (i) the $aTOF_{geo}$ is based on the total number of metal sites and the OER current density normalized to the geometric surface area. This approach does not take the change in ECSA upon activation into account. Note that this approach will overestimate the number of active sites, such that it provides a lower limit for the TOF. The method is therefore appropriate only for a comparison between films instead of providing absolute numbers. (ii) $aTOF_{ECSA}$ which is corrected for the change in electrochemical surface area after CV cycling.

The aTOF is calculated in the following manner,

$$aTOF_{geo} = j \cdot N_a / (4 \cdot F \cdot \Gamma_{bulk}) \quad (1)$$

$$aTOF_{ECSA} = aTOF_{geo} / (C_{activated} / C_{pristine}) \quad (2)$$

where, j, N_a, F, $\Gamma_{bulk,}$ $C_{activated,}$and $C_{pristine}$ represent current density, the Avogadro constant, the Faraday constant, the number of metal bulk sites, the adsorbate capacitance after 500 V cycles and the adsorbate capacitance of the pristine films. The number 4 represents the amount of electrons required to make one oxygen molecule.

Based on our previous work[7], the elemental concentration of Ni in NiO is determined to be (84±2)·10^{15} atoms/cm^2. The aTOF is calculated at 1.7 V vs RHE such that it resembles the overpotential more closely. Comparison of the $aTOF_{geo}$ trend with the overpotential based on the geometric surface area and obtained at 10 mA $cm^{-2}{}_{geo}$ (**Figure 4, S14**) shows similar results, validating the assumptions made for the aTOF calculations. Comparison of the $aTOF_{ESCA}$ to the corrected overpotential (**Figure 6, S15**) again shows the same trend, validating the corrected overpotential approach. Note that an increase in aTOF and a decrease in overpotential are indications for increased OER activity.

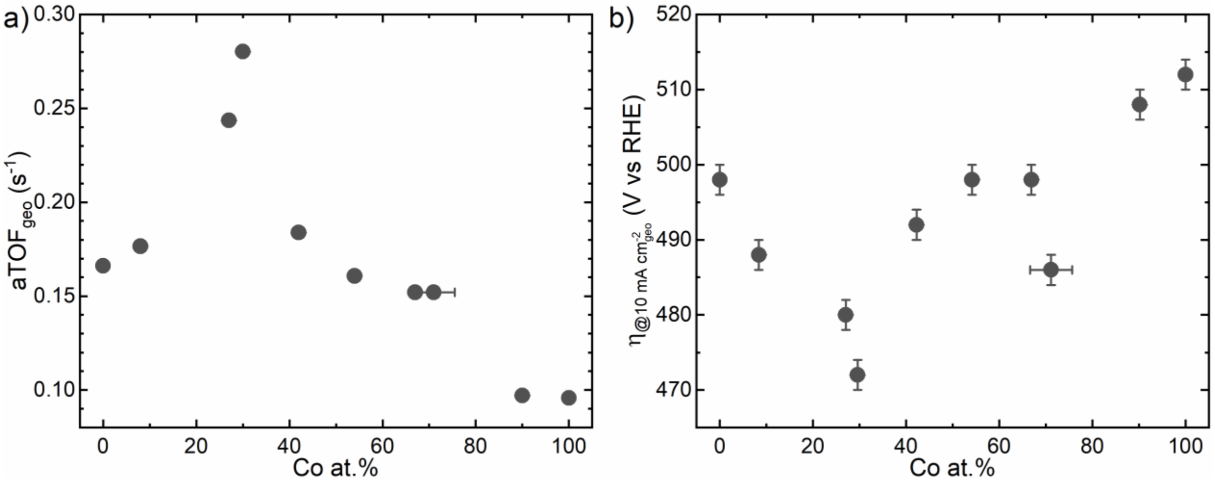

Figure S14 Comparison between the (a) $aTOF_{geo}$ calculated at 1.7 V vs RHE using equation (1) and (b) the overpotential per geometric surface area as presented at cycle 500 in **Figure 4a**.

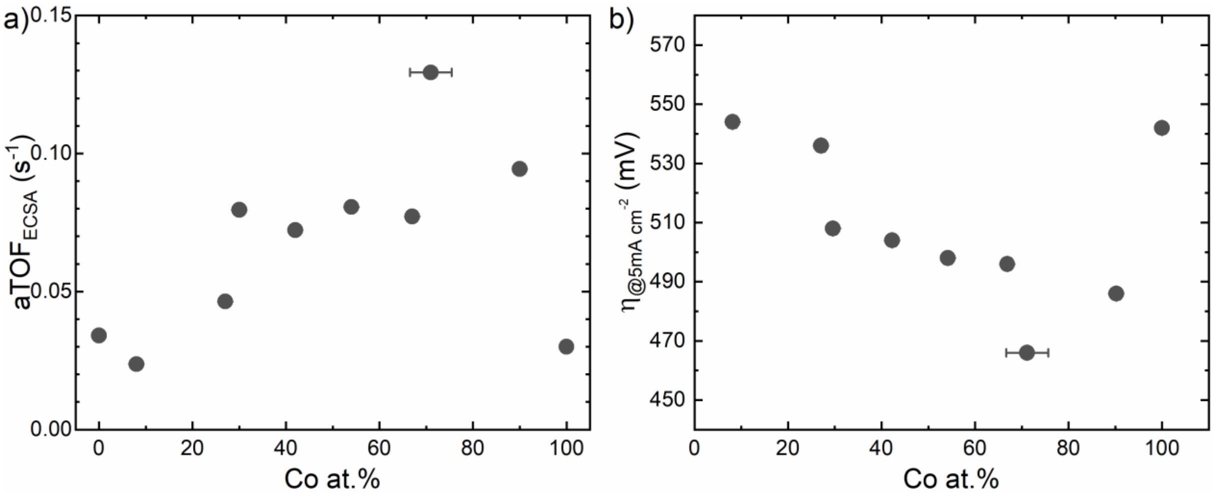

Figure S15 Comparison between the (a) $aTOF_{ECSA}$ calculated at 1.7 V vs RHE using equation (2) and (b) the overpotential at 5 mA cm^{-2}_{geo} corrected for the change in ECSA upon activation as presented in **Figure 6b**.

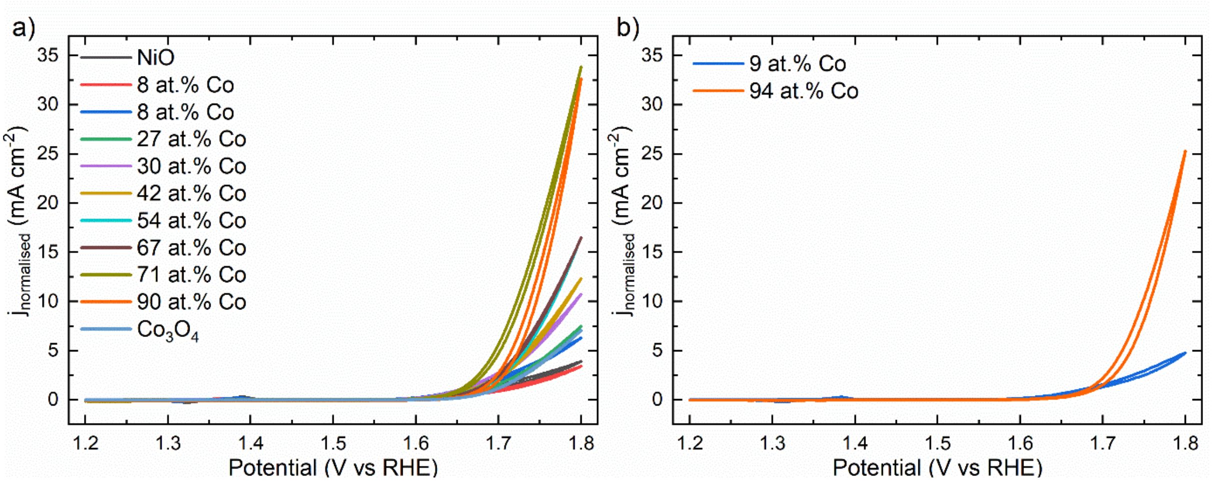

Figure S16 Current densities obtained after (a) 500 CV cycles and (b) 1000 cycles normalized to the change in electrochemical surface area as obtained from the change in adsorbate capacitance.

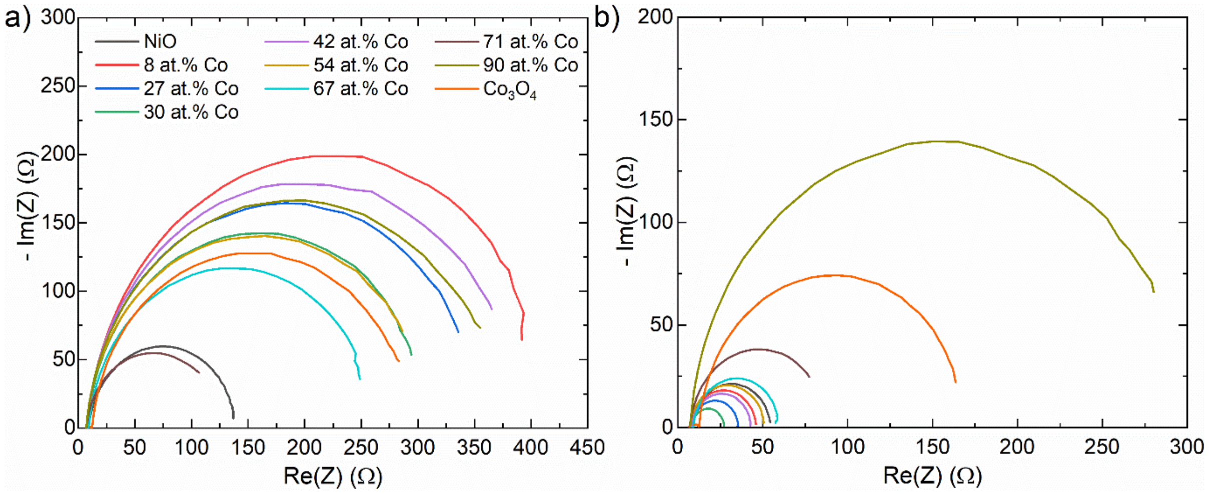

Figure S17 Electrochemical impedance spectra obtained at 1.6 V vs RHE for (a) pristine films and (b) after 500 CV cycles. The legend of Figure (a) is also valid for (b).

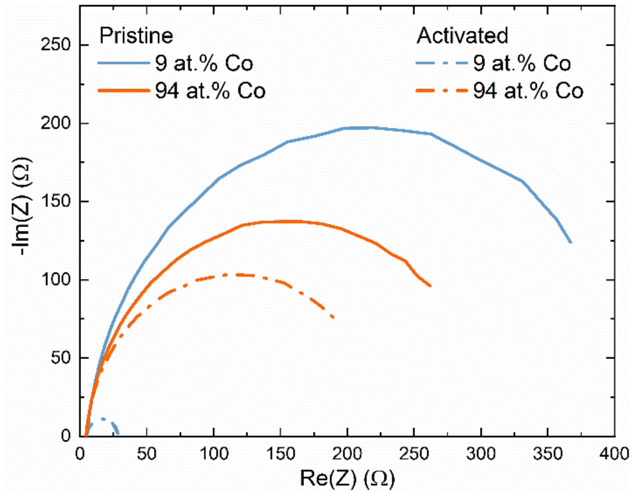

Figure S18 Electrochemical impedance spectra obtained at 1.6 V vs RHE for films before and after 1000 CV cycles.

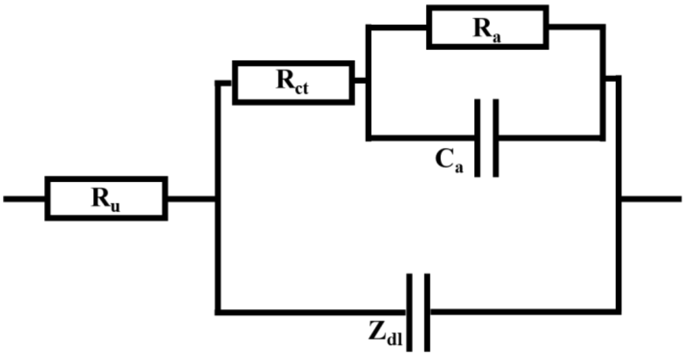

Figure S19 Equivalent electric circuit for determination of the adsorption capacitance (C_a), consisting of the uncompensated resistance (R_u), the charge transfer resistance (R_{ct}), adsorption resistance (R_a), and the double layer impedance (Z_{dl}). The circuit is adapted from Watzele *et al.*[26].

	Pristine films				
	R_u (Ω)	R_{ct} (Ω)	R_a (Ω)	Z_{dl} (μF)	C_a (μF)
**NiO**	8.66±0.03	40±3	87±3	78±1	58±3
**8 at.% Co**	7.26±0.06	29±5	371±7	80±3	43±3
**27 at.% Co**	7.51±0.03	96±8	242±8	143±1	63±3
**30 at.% Co**	8.10±0.02	95±7	197±7	149±1	68±4
**43 at.% Co**	7.96±0.03	116±9	255±9	148±1	67±4
**57 at.% Co**	6.99±0.02	91±7	202±7	200±2	96±5
**67 at.% Co**	9.05±0.03	75±6	168±6	21.3±2	113±6
**72 at.% Co**	6.89±0.02	27±2	88±3	780±10	440±10
**91 at.% Co**	6.92±0.03	118±9	243±9	126±1	75±5
**Co_3O_4**	11.77±0.08	79±10	195±11	160±4	80±7
**9 at.% Co (1000 CV cycles)**	5.26±0.02	0.9±0.1	317±7	125±1	54±2
**94 at.% Co (1000 CV cycles)**	4.45±0.02	71±6	215±6	185±2	89±5

Table S2 Obtained fitting results from the circuit presented in **Figure S19** for the EIS spectra of pristine films presented in **Figure S17a** and **S18**.

	Activated films					
	R_u (Ω)	R_{ct} (Ω)	R_a (Ω)	Z_{dl} (μF)	C_a (μF)	R_u (Ω)
**NiO**	8.66±0.03	8.70±0.02	25±1	19±1	269±0.3	280±30
**8 at.% Co**	7.26±0.06	6.56±0.04	1.18±0.04	37.5±0.2	15±1	321±3
**27 at.% Co**	7.51±0.03	6.95±0.06	1.02±0.06	27.1±0.1	5.4±7	328±3
**30 at.% Co**	8.10±0.02	7.93±0.01	9.1±0.9	10.1±0.9	293±6	240±30
**43 at.% Co**	7.96±0.03	8.09±0.02	15±2	19±2	256±5	170±20
**57 at.% Co**	6.99±0.02	7.02±0.01	16±1	27±1	301±5	190±10
**67 at.% Co**	9.05±0.03	9.00±0.02	20±2	30±2	346±5	220±20
**72 at.% Co**	6.89±0.02	6.92±0.02	0.16±0.02	61±2	980±20	510±20
**91 at.% Co**	6.92±0.03	7.06±0.02	74±7	207±7	201±2	77±4
**Co_3O_4**	11.77±0.08	9.5±0.2	3.2±2	150±1	1.3±0.1	255±2
**9 at.% Co (1000 CV cycles)**	5.26±0.02	5.03±0.01	14±1	9.1±0.8	530±6	610±90
**94 at.% Co (1000 CV cycles)**	4.45±0.02	4.44±0.02	65±5	150±5	277±3	123±7

Table S3 Obtained fitting results from the circuit presented in **Figure S19** for the EIS spectra of activated films presented in **Figure S17b** and **S18**.

References

[1] A. P. Grosvenor, M. C. Biesinger, R. S. C. Smart, N. S. McIntyre, *Surf. Sci.* **2006**, *600*, 1771.

[2] M. C. Biesinger, B. P. Payne, L. W. M. Lau, A. Gerson, R. S. C. Smart, *Surf. Interface Anal.* **2009**, *41*, 324.

[3] M. C. Biesinger, B. P. Payne, A. P. Grosvenor, L. W. M. Lau, A. R. Gerson, R. S. C. Smart, *Appl. Surf. Sci.* **2011**, *257*, 2717.

[4] D. Cabrera-German, G. Gomez-Sosa, A. Herrera-Gomez, *Surf. Interface Anal.* **2016**, *48*, 252.

[5] N. S. McIntyre, M. G. Cook, *Anal. Chem.* **1975**, *47*, 2208.

[6] D. B. Mitton, J. Walton, G. E. Thompson, *Surf. Interface Anal.* **1993**, *20*, 36.

[7] R. T. M. van Limpt, M. Lavorenti, M. A. Verheijen, M. N. Tsampas, M. Creatore, *J. Vac. Sci. Technol. A* **2023**, *41*, 32407.

[8] T. J. Frankcombe, Y. Liu, *Chem. Mater.* **2023**, *35*, 5468.

[9] L. Trotochaud, S. L. Young, J. K. Ranney, S. W. Boettcher, *J. Am. Chem. Soc.* **2014**, *136*, 6744.

[10] L. Trotochaud, J. K. Ranney, K. N. Williams, S. W. Boettcher, *J. Am. Chem. Soc.* **2012**, *134*, 17253.

[11] Z. Chen, L. Cai, X. Yang, C. Kronawitter, L. Guo, S. Shen, B. E. Koel, *ACS Catal.* **2018**, *8*, 1238.

[12] R. D. L. Smith, M. S. Prévot, R. D. Fagan, S. Trudel, C. P. Berlinguette, *J. Am. Chem. Soc.* **2013**, *135*, 11580.

[13] X. Deng, S. Öztürk, C. Weidenthaler, H. Tüysüz, *ACS Appl. Mater. Interfaces* **2017**, *9*, 21225.

[14] M. S. Burke, M. G. Kast, L. Trotochaud, A. M. Smith, S. W. Boettcher, *J. Am. Chem. Soc.* **2015**, *137*, 3638.

[15] S. Anantharaj, S. Kundu, S. Noda, *Nano Energy* **2021**, *80*, 105514.

[16] K. Kawashima, R. A. Márquez-Montes, H. Li, K. Shin, C. L. Cao, K. M. Vo, Y. J. Son, B. R. Wygant, A. Chunangad, D. H. Youn, G. Henkelman, V. H. Ramos-Sánchez, C. B. Mullins, *Mater. Adv.* **2021**, *2*, 2299.

[17] Y. Ou, L. P. Twight, B. Samanta, L. Liu, S. Biswas, J. L. Fehrs, N. A. Sagui, J. Villalobos, J. Morales-Santelices, D. Antipin, M. Risch, M. C. Toroker, S. W. Boettcher, *Nat. Commun.* **2023**, *14*, 7688.

[18] Y. Li, X. Lin, J. Du, *Inorg. Chem.* **2021**, *60*, 19373.

[19] Z. Wang, L. Wang, S. Liu, G. Li, X. Gao, *Adv. Funct. Mater.* **2019**, *29*, DOI 10.1002/adfm.201901051.

[20] C. Wei, S. Sun, D. Mandler, X. Wang, S. Z. Qiao, Z. J. Xu, *Chem. Soc. Rev.* **2019**, *48*, 2518.

[21] A. Ranade, M. Lao, R. H. M. Timmer, E. Zoethout, H. J. N. van Eck, M. N. Tsampas, *Adv. Mater. Interfaces* **2023**, *10*, 2300486.

[22] C. C. L. McCrory, S. Jung, I. M. Ferrer, S. M. Chatman, J. C. Peters, T. F. Jaramillo, *J. Am. Chem. Soc.* **2015**, *137*, 4347.

[23] R. Zhang, G. van Straaten, V. di Palma, G. Zafeiropoulos, M. C. M. van de Sanden, W. M. M. Kessels, M. N. Tsampas, M. Creatore, *ACS Catal.* **2021**, *11*, 2774.

[24] S. S. Jeon, P. W. Kang, M. Klingenhof, H. Lee, F. Dionigi, P. Strasser, *ACS Catal.* **2023**, *13*, 1186.
[25] A. S. Batchellor, S. W. Boettcher, *ACS Catal.* **2015**, *5*, 6680.
[26] S. Watzele, P. Hauenstein, Y. Liang, S. Xue, J. Fichtner, B. Garlyyev, D. Scieszka, F. Claudel, F. Maillard, A. S. Bandarenka, *ACS Catal.* **2019**, *9*, 9222.
[27] S. Anantharaj, P. E. Karthik, S. Noda, *Angew. Chemie Int. Ed.* **2021**, *60*, 23051.
